# Artificial neural network analysis of Jeffrey hybrid nanofluid with gyrotactic microorganisms for optimizing solar thermal collector efficiency

**DOI:** 10.1038/s41598-025-88877-6

**Published:** 2025-02-08

**Authors:** Anup Kumar, Bhupendra K. Sharma, Bandar Almohsen, Laura M. Pérez, Kamil Urbanowicz

**Affiliations:** 1 Department of Mathematics, Birla Institute of Technology and Science Pilani, Rajasthan, India; 2 Department of Mathematics, College of Science, King Saud University, P.O. Box 2455, Riyadh, 11451 Saudi Arabia; 3 Departamento de Ingeniería Industrial y de Sistemas, Universidad de Tarapacá, Casilla 7D, Arica, 1000000 Chile; 4 Faculty of Mechanical Engineering and Mechatronics, West Pomeranian University of Technology in Szczecin, Al. Piastów 17, 70-310 Szczecin, Poland

**Keywords:** Solar energy, Graphene and Silver nanoparticles, Gyrotactic microorganisms, Electro-magneto-hydrodynamic, Engineering, Mathematics and computing, Nanoscience and technology

## Abstract

This article investigates solar energy storage due to the Jeffrey hybrid nanofluid flow containing gyrotactic microorganisms through a porous medium for parabolic trough solar collectors. The mechanism of thermophoresis and Brownian motion for the graphene and silver nanoparticles are also encountered in the suspension of water-based heat transfer fluid. The gyrotactic microorganisms have the ability to move in an upward direction in the nanofluid mixture, which enhances the nanoparticle stability and fluid mixing in the suspension. Mathematical modeling of the governing equations uses the conservation principles of mass, momentum, energy, concentration, and microorganism concentration. The non-similar variables are introduced to the dimensional governing equations to get the non-dimensional ordinary differential equations. The Cash and Carp method is implemented to solve the non-dimensional equations. The artificial neural network is also developed for the non-dimensional governing equations using the Levenberg Marquardt algorithm. Numerical findings corresponding to the diverse parameters influencing the nanofluid flow and heat transfer are presented in the graphs. The thermal profiles are observed to be enhanced with the escalation in the Darcy and Forchheimer parameters. And the Nusselt number enhances with the escalation in the Deborah number and retardation time parameter. Entropy generation reduces with an enhancement in Deborah number and retardation time parameter. Solar energy is the best renewable energy source. It can fulfill the energy requirements for the growth of industries and engineering applications.

## Introduction

In the modern era, total energy consumption is rising due to high living standards, growth in industrialization, and increasing population. Renewable energy sources dominate the enormous amount of energy used in the world’s economy. Solar energy is the best alternative renewable energy source, which supplies clean and sustainable energy to industrial and technological societies. Among the various solar technologies, parabolic trough solar collectors are primarily used for industrial and engineering purposes to produce high-temperature steams. The parabolic trough reflects and concentrates the sun’s radiation about the receiver tube placed at the focus of the trough that can absorb the concentrated solar energy and store it with the help of heat transfer fluids, which is helpful in various thermal engineering applications, including electricity generation, industrial heat processes, solar heating and cooling, and solar desalination. Nanofluids exhibit a non-Newtonian nature because of suspended nanoparticles in conventional fluids. Adding nanoparticles to the base fluids will significantly affect the base fluids’ viscosity. Therefore, the viscosity varies with the concentration of the nanoparticles, which makes the non-Newtonian behavior of the fluids. This non-Newtonian behavior of nanofluid is experimentally and theoretically validated by the researchers in past studies^1–3^. Multiple non-Newtonian fluids exist in nature and have various applications in engineering and technology. Several researchers did different studies on the various type of non-Newtonian fluids^4–11^. Among these, the Jeffrey fluid model is notable over various rheological fluid models because it has the shear thinning property of the liquids and captures the stress relaxation property. The non-Newtonian characteristic of the Jeffrey hybrid nanofluid means its viscosity decreases as shear stress increases, making it shear-thinning. This reduces resistance to fluid flow as it speeds up, enhancing heat transfer efficiency. Tripathi et al.^12^ investigated the peristaltic Jeffrey fluid flowing through the tube. The study of an electrically conducting Jeffrey fluid for the stagnation point flow with partial surface slip conditions was done by Das et al.^13^. Ahmad and Ishak^14^ explored the heat transmission of the Jeffrey fluid along a stretched sheet for mixed convection flow through a porous material. Zeeshan et al.^15^ discussed the magnetohydrodynamics bio-bi-phase flow effects on the peristaltic transport of Jeffrey nanofluid flowing in a duct with uniformly distributed rigid particles. Vaidya et al.^16^ conducted the study of heat transport for magneto-hydrodynamic peristaltic flow along an asymmetrical tapered channel. Ge-gile^17^ studied the two-phase motion of multi-layer hydrodynamic fluid through a porous medium. The influences of Ohmic heating and thermal radiation for the peristaltic transport of magnetized Jeffery fluid were studied by Hussain et al.^18^. Sharma and Gandhi did the^19^ entropy optimization of tetra hybrid Jeffrey nanofluid flow in a bifurcation artery with Hall effects.

The Ohmic heating technology has various advantages like quick achievement of temperature, low heat loss, low maintenance cost, etc. This process has a variety of applications, like the creation of electricity, the production of energy, nuclear reactors, the cooling of atomic reactors, etc. The authors did several research studies in this field to study such phenomena. Flow from an exponential elongating surface with the Soret and Dufour effects are examined in the work of Hayat et al.^20^. Hayat et al.^21^ also described the mixed convective peristaltic fluid flow driven from the flexible walls with Ion and Hall slip currents. Cattaneo-Christov heat flux with two-layered stratification and an exponential heat source in nanofluid flow across a melting surface was analyzed by Mehanthesh et al.^22^. Soid et al.^23^ investigated the magnetohydrodynamic viscous fluid flow with Ohmic heating along an elongating sheet. Entropy generation with Ohmic heating and hall effects in peristaltic motion of nanofluid through a symmetric channel was estimated by Abbasi et al.^24^. Yousif et al.^25^ attempted MHD carreau nanofluid momentum and heat transfer over a spontaneously elongation surface with thermal radiation. Rasheed et al.^26^ studied the thermally radiative mixed convection magnetohydrodynamic Jeffrey nanofluid flow through a vertically stretchable cylinder. Dadhich et al.^27^ analyzed the utilization of heat source on the flow of a thermally radiative Jeffrey fluid containing nanoparticles. Heat transfer analysis of the micro planar fluid over a curved elongating surface was done by Qian et al.^28^. Entropy generation analysis was conducted by Sharma et al.^29^ for the electrically conductive fluid (Blood) flow containing nanoparticles through an irregularly stenosed artery with catheter thrombosis. Rehman et al.^30^ did the multiple-physics simulations of magnetohydrodynamic Carreau fluid flow with the Soret and Dufour phenomena under the thermal radiation and Darcy–Forchheimer effects. The influence of a diagonal magnetic field on unsteady mixed convective stagnation point flow across a porous stretched sheet with radiative heat transmission was investigated by Chen et al.^31^. The effect of electroosmotic pressure on the hybrid nanofluid’s passage through the microchannel containing ferri- and para-magnetic nanoparticles was explored by Ramzan et al.^32^. The impact of the generated magnetic field on Darcy-Forchheimer nanofluid flows incorporating carbon nanotubes with homogeneous-heterogeneous reactions was studied by^33^.

The review and feasibility of multiple methods to enhance heat transmission in parabolic trough receivers, which includes evacuated receivers with/without inserts and inserts with nanofluids as investigated by Sandeep and Arunachala^34^. Characterizing the dimpled and protruded absorbers using thermo-hydraulic methods in solar thermal collectors was studied by Chauhan et al.^35^. Nagpal et al.^36^ did the numerical simulation to analyze the assessment of parabolic trough collectors’ performance through energy and exergy analysis, focusing on second law efficiency, which necessitates exploring diverse system and operational parameters. Improving heat transfer within these solar collectors can be effectively achieved by harnessing thermal radiation and leveraging nanotechnological advancements in energy conversion, as studied by Reddy et al.^37^. Various design and operating parameters that influence the efficacy of solar heaters with latent heat storage were analyzed by Sharma et al.^38^. Varun et al.^39^ reviewed and explored the sustainable mechanisms to promote the widespread use of domestic solar cooking. Gaur et al.^40^ classified the performance parameters of Solar dryers to improve the effectiveness of the desired drying process. An evaluation of the bifacial photovoltaic thermal dryer’s performance coupled with heat storage was studied by Sehrawat et al.^41^. Sharma et al.^42^ attempted the phase change material for latent heat storage units intended for thermal appliances of low temperature. Applications of Darcy-Forchheimer theory to the flow and heat transfer of Reiner-Philippoff nanofluid lubrication with thermal and solutal slip implications of tribological coatings are made by Rehman et al.^43^. The substantial role of Darcy-Forchheimer with incorporated hybrid nanoparticles on heat conduction for the dusty nanofluid flow was studied by Ali et al.^44^.

Gyrotactic microorganisms propelled by the torque generated from gravitational and viscous forces are called motile gyrotactic microorganisms. Also, motile gyrotactic microorganisms can swim and respond to flow gradients, further enhancing heat transfer. These microorganisms create flow patterns, stabilize nanoparticles, turbulence, and localized agitation within the fluid, improving convective heat transfer. It combines shear-thinning behavior and enhanced thermal conductivity with convective heat transfer enhancement, making it a promising solution for efficient heat transfer. Gyrotactic microorganisms travel in a swing motion due to the bioconvection process, improving nanoparticle stability as explored by Avramenko and Kuznetsov^45^. A theoretical study of bioconvection’s Darcy-Forchheimer flow of Casson fluid with a chemical reaction effect was presented by Rehman et al.^46^. An investigation was conducted by Sharma et al.^47^ for the Jeffrey fluid undergoing electro-magneto-hydrodynamic flow with the gyrotactic microorganisms. The reactive chemical process in unsteady bioconvective magneto Williamson nanofluid flow through wedge with nonlinear thermal radiation under the Darcy-Forchheimer paradigm was studied by Rehman et al.^48^. Sharma et al.^49^ explored magnetohydrodynamic nanofluid flow with gyrotactic microorganisms through porous media. Khanduri et al.^50^ analyzed the impact of gyrotactic microorganisms on hybrid nanofluid’s electroosmotic flow in a bifurcation artery having stenosis in both the parent and daughter arteries. Aspects of Darcy-Forchheimer on unsteady bioconvection flow of Reiner-Philippoff nanofluid along a wedge containing swimming microbes were studied by Rehman et al.^51^. The effect of bioconvection on MHD Carreau fluid’s Darcy-Forchheimer flow with Arrhenius activation energy was examined by Rehman et al.^52^. Unsteady bioconvective radiative Casson nanofluid flow between two concentric stretched cylinders with different thermal conductivity and viscosity was discussed by Alharbi et al.^53^. Arrhenius activation energy and chemical reaction in a Darcy-Forchheimer permeable medium driving buoyancy-driven bioconvective Casson nanofluid flow over a vertical stretching cylinder was examined by Zhao et al.^54^. An incomparable remedy for dissipative Eyring-Powell nanofluid flow across a nonlinear stretching surface for an inclined magnetic field explored by Liu et al.^55^.

Artificial neural networks (ANNs) are widely used in various engineering fields, particularly in modeling complicated physical processes such as those encountered in thermal engineering. Their use speeds up the modeling process and increases the viability and appeal of solar-energy applications by identifying ideal solutions. Artificial neural networks excel at identifying critical information patterns within complex multidimensional data fields. Artificial neural networks are a powerful non-linear analog approach in the computational intelligence domain. Comprehensive evaluations emphasize their significance in thermal engineering, specifically for renewable energy. Artificial neural networks are used by Yaici et al.^56^ to evaluate the solar energy storage intended for home space heating. The artificial neural network approach was used by Qureshi et al.^57^ to predict precise and efficient models of hybrid nanofluids in heat transfer. The study of Ermis et al.^58^ proposed an artificial neural network (ANN) utilizing feed-forward backpropagation to analyze the heat transmission in a finned-tube latent heat thermal energy storage device during a phase change. Sharma et al.^59^ made an artificial network for studying heat transfer due to Jeffrey fluid for solar thermal collectors.

The existing technology problems have not addressed the artificial neural networks for heat transfer of the solar energy storage due to non-Newtonian Jeffrey hybrid nanofluid flow with thermophoretic diffusion and Brownian motion of nanoparticles under suspended gyrotactic microorganisms with Darcy Forchheimer model. Although the related technologies proposed to solve this type of invention are limited to the implications of Newtonian fluids as heat transfer fluids. However, various advanced non-Newtonian types of heat transfer fluids are available in the literature. Also, previous inventions introduced nanoparticles in the heat transfer fluid known as nanofluids to enhance the conductive properties of conventional fluids. However, the nanoparticle stabilities in suspension are a significant issue in the context of nanofluids. The upward suspension of the gyrotactic microorganism increases the convective heat transmission and enhances the nanoparticle’s stability in the fluid flow. Our proposed invention describes the heat transmission due to electro-magneto-hydrodynamic Jeffrey’s hybrid nanofluid flow in solar collectors with thermophoresis and Brownian motion of nanoparticles influenced by the suspended gyrotactic microorganisms through Darcy Forchheimer porous medium. An advanced hybrid nanofluid is selected for effective heat transmission, with graphene and silver as the hybrid nanoparticles with the water-based fluid. Novelties and the main objectives of this invention are pointed out as follows:

- Electromagnetohydrodynamic Jeffrey nanofluid model is considered for the heat transmission to enhance the performance of solar trough collectors.
- The contribution of Joule heating with Darcy Forchheimer porous medium is analyzed in the heat transfer for solar collectors.
- The heat transfer augmentation due to the suspension of Graphene and Silver hybrid nanoparticles is investigated in water-based heat transfer fluid.
- The suspended gyrotactic microorganisms is taken to enhance the stability of hybrid nanoparticles in fluid flow.
- Artificial neural network is also applied to the developed mathematical model for the different input parameters.
- Darcy Forchheimer’s law is incorporated to model the porous effect in the fluid flow.
- The entropy generation is studied for estimating the thermal losses in the heat transmission for the Jeffrey hybrid nanofluid flow.

The proposed article aims to overcome the challenges mentioned above by introducing a specific solution involving Jeffrey non-Newtonian hybrid nanofluids and gyrotactic microorganisms in solar collectors. Gyrotactic microorganisms exhibit upward movement due to torque, contributing to the upswing suspension and fluid mixing. This motion also preserve the stability of hybrid nanoparticles in the heat transfer fluid. Graphene and Gold nanoparticles were chosen as hybrid nanoparticles. This selection aims to improve the conductive properties of the conventional fluid and enhance heat transfer efficiency. In summary, the invention addresses the challenges by incorporating advanced non-Newtonian hybrid nanofluids, leveraging the motion of motile gyrotactic microorganisms for improved fluid stability, and carefully selecting hybrid nanoparticles to enhance heat transfer properties. The main goal is to optimize solar collectors’ thermal performance by addressing the suspension of gyrotactic microorganisms in hybrid nanofluids.

## Mathematical formulation

**Fig. 1:**
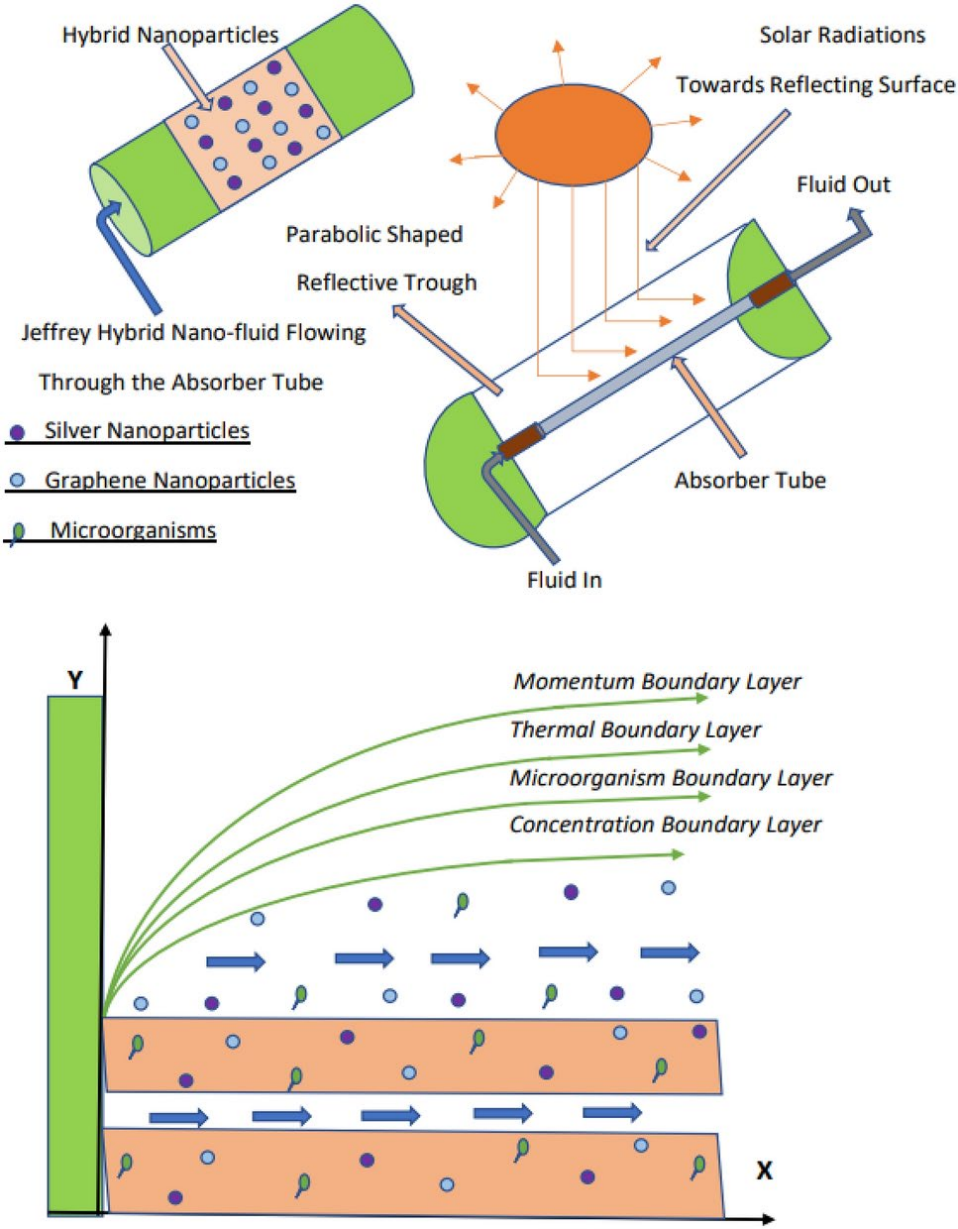
Physical representation of the fluid model.

This study considers a time-independent, two-dimensional, incompressible Jeffrey boundary layer hybrid nanofluid flow. The nanofluid consists of water as the heat transfer fluid along with Silver and Graphene nanoparticles undergoing thermophoretic diffusion and Brownian motion. Additionally, the fluid contains motile gyrotactic microorganisms, and the flow occurs through a Darcy Forchheimer porous medium along a horizontal elongating surface. The negligible influence of the induced magnetic field is assumed, characterized by a low Reynolds number ($Re<< 1$). The current density generated in the fluid due to external electric and magnetic fields is defined as **J**=$\sigma ({\textbf {E}}+{\textbf {V}}\times {\textbf {B}})$ where **E** is the electric field, **V** denotes the velocity, and **B** is the applied magnetic field. The magnetic field alone can induce sufficient current density for fluids with high electrical conductivity, obviating the need for an electric field. In contrast, fluids with low electrical conductivity require an external electric field to control fluid flow effectively. Here, the Lorentz force due to the effect of the magnetic field is expressed as ${\textbf {F}}=\sigma ({\textbf {V}}\times {\textbf {B}}) \times {\textbf {B}}$. It is presumed that the suspended nanoparticles do not affect the gyrotactic microbe’s swimming direction or speed. The governing equations for the nanofluid flow are modeled using boundary layer assumptions. Figure 1 illustrates the physical representation of the nanofluid model.

Steps of the mathematical formulation to simulate the fluid flow model are:

- The governing equations are derived with boundary layer assumption for the non-Newtonian Jeffrey nanofluid model by introducing different source terms like Joule heating, viscous dissipation, porous medium, thermophoretic diffusion, and Brownian motion along with bioconvection mechanism of gyrotactic microorganism in the presence of solar radiation.
- Considering the mixture of Silver and Graphene hybrid nanoparticles with water as the heat transfer fluid.
- Non-similar variables are utilized to get the dimensionless governing equations to obtain the dimensionless form of governing equations.
- The resulting equations are then numerically solved by the Cash and Carp numerical method.
- The different results for the various defaults of the influential physical parameters are plotted for the thermal profiles, Nusselt number, and entropy generation using line graphs, surface plots, and contour plots.

The stress tensor components for Jeffrey hybrid nanofluid flow is:

$$\begin{aligned} \begin{aligned} {{\varvec{T}} = -\textit{P} \textit{I}+\textit{S}}, \end{aligned} \end{aligned}$$

where, expression of *S* is formulated as:

2.1 $$\begin{aligned} {\textbf {S}}=\frac{\mu _{hnf}}{1+\lambda _{1}}\bigg [{\textbf {A}}+\lambda _{2}\frac{d{\textbf {A}}}{dt}\bigg ], \end{aligned}$$

where, *A* is the Rivlin Erection tensor written as:

**A** = $\nabla V + (\nabla V)^{\prime }$, where, $'$ is the transpose.

Equations governing the Jeffrey hybrid nanofluid flow are^60,61^:

2.2 $$\begin{aligned} & \frac{\partial {u}}{\partial x}+\frac{\partial {v}}{\partial y}=0, \end{aligned}$$

2.3 $$\begin{aligned} & \quad u\frac{\partial u}{\partial x}+v\frac{\partial u}{\partial y}=\frac{\nu _{hnf}}{(1+\lambda _{1})}\bigg [\frac{\partial ^{2} u}{\partial y^2}+\lambda _{2}\bigg (u\frac{\partial ^{3} u}{\partial x \partial y^2}+\frac{\partial u}{\partial y}\frac{\partial ^{2} u}{\partial x \partial y}+v\frac{\partial ^{3} u}{\partial y^3} -\frac{\partial u}{\partial x}\frac{\partial ^{2} u}{\partial y^2}\bigg )\bigg ] - \frac{\nu _{hnf}}{K_1}u -\frac{c_b}{\sqrt{K_1}} u^2 -\frac{\sigma _{hnf}}{\rho _{hnf}}(B^2u-EB), \end{aligned}$$

2.4 $$\begin{aligned} & \quad u\frac{\partial T}{\partial x}+ v\frac{\partial T}{\partial y}=\frac{\kappa _{hnf}}{(\rho Cp)_{hnf}}\frac{\partial ^2 T}{\partial y^{2}}+\frac{16 \sigma T_\infty ^3}{3 \kappa ^* (\rho c_p)_{hnf}} \frac{ \partial ^2 T}{\partial y^{2}} +\tau \bigg (D_{B}\frac{\partial C}{\partial y}\frac{\partial T}{\partial y}+\frac{D_{T}}{T_{\infty }}\bigg (\frac{\partial T}{\partial y}\bigg )^{2}\bigg )+\frac{\mu _{hnf}}{({\rho Cp})_{hnf}(1+\lambda _{1})}\bigg [\bigg (\frac{\partial u}{\partial y}\bigg )^{2}\nonumber \\ & \quad +\lambda _{2} \bigg (u \frac{\partial u}{\partial y} \frac{\partial ^{2} u}{\partial x \partial y}+v \frac{\partial u}{\partial y} \frac{\partial ^{2} u}{\partial y^{2}}\bigg )\bigg ] +\frac{\sigma _{hnf}}{({\rho Cp})_{hnf}}(u B(x)-E(x))^{2}, \end{aligned}$$

2.5 $$\begin{aligned} & \quad u\frac{\partial C}{\partial x}+ v\frac{\partial C}{\partial y}=\frac{D_{T}}{T_{\infty }}\frac{\partial ^2 T}{\partial y^{2}}+D_{B}\frac{\partial ^{2} C}{\partial y^{2}}, \end{aligned}$$

2.6 $$\begin{aligned} & \quad u\frac{\partial N}{\partial x}+ v\frac{\partial N}{\partial y}+\bigg [\frac{\partial }{\partial y} \bigg (N \frac{\partial C}{\partial y}\bigg )\bigg ] \frac{bW_c}{C_w-C_\infty } =D_{m} \frac{\partial ^{2} N}{\partial y^{2}}. \end{aligned}$$

Associated boundary conditions are:

2.7 $$\begin{aligned} & u=U_{s}(x),\quad v=0,\quad T=T_{s},\quad C=C_{s}, \quad N=N_{s}, \quad at \quad y=A(x+c)^{\frac{1-n}{2}}, \end{aligned}$$

2.8 $$\begin{aligned} & \quad u\rightarrow 0, \quad T \rightarrow T_{\infty },\quad C \rightarrow C_{\infty },\quad N \rightarrow N_{\infty }, \quad as \quad y \rightarrow \infty . \end{aligned}$$

where $B(x)=B_{0}(x+c)^\frac{n-1}{2}$ is the magnetic field strength in normal direction, $U_{s}(x)=b(x+b)^{n},$ is the elongation velocity at $y=A(x+c)^\frac{(1-n)}{2}$, and *n* is the power index.

Mathematical relations to estimate the physical properties of the hybrid nanofluid:

Dynamic viscosity

$$\begin{aligned} \mu _{h n f}=\frac{\mu _f}{\left( 1-\phi _{1}\right) ^{2.5}\left( 1-\phi _{2}\right) ^{2.5}}, \end{aligned}$$

Density

$$\begin{aligned} \rho _{h n f}=\left( 1-\phi _{1}\right) \left[ \left( 1-\phi _{2}\right) \rho _f+\phi _{2} \rho _{2}\right] +\phi _{1} \rho _{1}, \end{aligned}$$

Specific heat capacity

$$\begin{aligned} \begin{aligned} \left( \rho c_p\right) _{h n f}=\left( 1-\phi _{1}\right) \left[ \left( 1-\phi _{2}\right) \left( \rho c_p\right) _f+\phi _{2}\left( \rho c_p\right) _{2}\right] +\phi _{1}\left( \rho c_p\right) _{1}, \end{aligned} \end{aligned}$$

Thermal conductivity

$$\begin{aligned} k_{h n f}=\frac{k_{1}+2 k_{n f}-2 \phi _{1}\left( k_{n f}-k_{1}\right) }{k_{1}+2 k_{n f}+\phi _{1}\left( k_{n f}-k_{1}\right) } \times \left( k_{n f}\right) , \end{aligned}$$

where

$$\begin{aligned} k_{n f}=\frac{k_{2}+2 k_f-2 \phi _{2}\left( k_f-k_{2}\right) }{k_{2}+2 k_f+\phi _{2}\left( k_f-k_{2}\right) } \times \left( k_f\right) , \end{aligned}$$

Electrical conductivity

$$\begin{aligned} \sigma _{h n f}=\frac{\sigma _{1}+2 \sigma _{n f}-2 \phi _{1}\left( \sigma _{n f}-\sigma _{1}\right) }{\sigma _{1}+2 \sigma _{n f}+\phi _{1}\left( \sigma _{n f}-\sigma _{1}\right) } \times \left( \sigma _{n f}\right) , \end{aligned}$$

where

$$\begin{aligned} \sigma _{n f}=\frac{\sigma _{2}+2 \sigma _f-2 \phi _{2}\left( \sigma _f-\sigma _{2}\right) }{\sigma _{2}+2 \sigma _f+\phi _{2}\left( \sigma _f-\sigma _{2}\right) } \times \left( \sigma _f\right) . \end{aligned}$$

## Non-similar analysis

Non-similarity solutions are renowned in fluid mechanics for their precision and effectively reducing the independent variables. These solutions typically emerge as asymptotic solutions, offering valuable physical characteristics of complex fluid scenarios. They encapsulate the current dynamic, physical, and thermal scenarios and elucidate their effects. The Non-similar variables employed to derive the non-dimensional governing equations are:

$$\begin{aligned} \begin{aligned} \xi&=\frac{x+c}{l}, \quad \zeta =\sqrt{\frac{(n+1) b(x+c)^{n-1}}{2 v_f}} y, \quad u=b(x+c)^n \frac{\partial f}{\partial \zeta }(\xi , \zeta ), \quad \theta (\xi , \zeta )=\frac{T-T_{\infty }}{\left( T_s-T_{\infty }\right) }, \quad \phi (\xi , \zeta )=\frac{C-C_{\infty }}{\left( C_s-C_{\infty }\right) }, \\&\chi (\xi , \zeta )=\frac{N-N_{\infty }}{\left( N_s-N_{\infty }\right) }, \quad v =-\sqrt{\frac{v_f(n+1) b(x+c)^{n-1}}{2}}\left( \frac{2 \xi }{n+1} \frac{\partial f}{\partial \xi }(\xi , \zeta )+f(\xi , \zeta )+\frac{n-1}{n+1} \zeta \frac{\partial f}{\partial \zeta }(\xi , \zeta )\right) \end{aligned} \end{aligned}$$

By utilizing the above non-similar variables to get the dimensionless governing equations:

3.1 $$\begin{aligned} & \frac{\partial ^4 f}{\partial \zeta ^4}\bigg (\xi \frac{\partial f}{\partial \xi }+\frac{n+1}{2}f+\frac{n-1}{2}\zeta \frac{\partial f}{\partial \zeta }\bigg )=\frac{2(1+\beta _1)}{A_1 \beta _2}\bigg [-\frac{A_1 K_p}{A_2 (n+1)} \frac{\partial f}{\partial \zeta }-\frac{Fr}{n+1} \bigg (\frac{\partial f}{\partial \zeta }\bigg )^2- \frac{A_3 M}{A_2 (n+1)} \bigg (\frac{\partial f}{\partial \zeta }-E_1\bigg )\bigg ]\nonumber \\ & \quad +\frac{1}{\beta _2}\frac{\partial ^3 f}{\partial \zeta ^3}+ (n-1)\frac{\partial f}{\partial \zeta }\frac{\partial ^3 f}{\partial \zeta ^3}+\xi \frac{\partial f}{\partial \zeta } \frac{\partial ^4 f}{\partial \xi \partial \zeta ^3}+n\bigg (\frac{\partial ^2 f}{\partial \zeta ^2}\bigg )^2+\xi \frac{\partial ^2 f}{\partial \zeta ^2} \frac{\partial ^3 f}{\partial \zeta ^2 \partial \xi }-\xi \frac{\partial ^2 f}{\partial \xi \partial \zeta } \frac{\partial ^3 f}{\partial \zeta ^3}-\frac{2(1+\beta _1)}{A_1 \beta _2}\bigg [\frac{n}{n+1}\bigg (\frac{\partial f}{\partial \zeta }\bigg )^2\nonumber \\ & \quad +\frac{\xi }{n+1}\frac{\partial f}{\partial \zeta } \frac{\partial ^2 f}{\partial \zeta \partial \xi }-\frac{\xi }{n+1}\frac{\partial ^2 f}{\partial \zeta ^2}\frac{\partial f}{\partial \xi }-\frac{f}{2}\frac{\partial ^2 f}{\partial \zeta ^2}-\frac{n-1}{2(n+1)} \zeta \frac{\partial ^2 f}{\partial \zeta ^2}\frac{\partial f}{\partial \zeta }\bigg ] \end{aligned}$$

3.2 $$\begin{aligned} & \quad \bigg (1+\frac{4}{3}Rd\bigg )\frac{\partial ^2 \theta }{\partial \zeta ^2}=\frac{2}{n+1}\frac{A_1 A_5}{A_2 A_4} \xi \frac{\partial f}{\partial \zeta }\frac{\partial \theta }{\partial \xi }- \frac{A_1 A_5}{A_2 A_4}\frac{\partial \theta }{\partial \zeta }\bigg (\frac{2\xi }{n+1} \frac{\partial f}{\partial \xi }+f+\frac{n-1}{n+1}\zeta \frac{\partial f}{\partial \zeta }\bigg )-\frac{A_3 A_1}{A_2 A_4}\frac{2}{n+1}Pr Ec M..\nonumber \\ & \quad ..\bigg (\frac{\partial f}{\partial \zeta }-E_1\bigg )^2-\frac{A_5}{A_4}Pr Nb \frac{\partial \phi }{\partial \zeta }\frac{\partial \theta }{\partial \zeta }-\frac{A_5}{A_4}Pr Nt \bigg (\frac{\partial \theta }{\partial \zeta }\bigg )^2-\frac{A_1}{A_4}\frac{Pr Ec}{1+\beta _1}\bigg [\bigg (\frac{\partial ^2 f}{\partial \zeta ^2}\bigg )^2+\beta _2 \bigg [\bigg (\frac{\partial ^2 f}{\partial \zeta ^2}\bigg )^2\frac{\partial f}{\partial \zeta }+\xi \frac{\partial ^3 f}{\partial \zeta ^2 \partial \xi }\frac{\partial f}{\partial \zeta }\frac{\partial ^2 f}{\partial \zeta ^2}\nonumber \\ & \quad -\frac{n+1}{2}\bigg (\frac{2\xi }{n+1} \frac{\partial f}{\partial \xi }+f+\frac{n-1}{n+1}\zeta \frac{\partial f}{\partial \zeta }\bigg )\frac{\partial ^2 f}{\partial \zeta ^2}\frac{\partial ^3 f}{\partial \zeta ^3} \bigg ]\bigg ],~~ \end{aligned}$$

3.3 $$\begin{aligned} & \quad \frac{\partial ^2 \phi }{\partial \zeta ^2}+\frac{Nt}{Nb}\frac{\partial ^2 \theta }{\partial \zeta ^2}=\frac{A_1}{A_2}\frac{2}{n+1}Le \xi \frac{\partial f}{\partial \zeta }\frac{\partial \phi }{\partial \xi }-\frac{A_1}{A_2}Le\bigg (\frac{2\xi }{n+1} \frac{\partial f}{\partial \xi }+f+\frac{n-1}{n+1}\zeta \frac{\partial f}{\partial \zeta }\bigg )\frac{\partial \phi }{\partial \zeta }, \end{aligned}$$

3.4 $$\begin{aligned} & \quad \frac{\partial ^2 \chi }{\partial \zeta ^2}=\frac{A_1}{A_2}\frac{2}{n+1}Lb \xi \frac{\partial f}{\partial \zeta }\frac{\partial \chi }{\partial \xi }-\frac{A_1}{A_2}Lb\bigg (\frac{2 \xi }{n+1} \frac{\partial f}{\partial \xi }+f+\frac{n-1}{n+1}\zeta \frac{\partial f}{\partial \zeta }\bigg )\frac{\partial \chi }{\partial \zeta }+Pe\frac{\partial \phi }{\partial \zeta }\frac{\partial \chi }{\partial \zeta }+N_{\delta }\bigg (1+\frac{1}{N_{\delta }}\bigg )Pe \frac{\partial ^2 \phi }{\partial \zeta ^2}. \end{aligned}$$

Boundary conditions in dimensionless form:

3.5 $$\begin{aligned} & \frac{2 \xi }{n+1} f^\prime (\xi , \zeta )+f(\xi , \zeta )+\frac{n-1}{n+1} \zeta f^\prime (\xi , \zeta )=0, \quad f^{\prime }(\xi ,\zeta )=1, \quad \theta (\xi ,\zeta )=1, \quad \phi (\xi ,\zeta )=1, \quad \chi (\xi ,\zeta )=1 \quad \text{ at } \zeta =0, \end{aligned}$$

3.6 $$\begin{aligned} & \quad f^{\prime }(\xi ,\zeta ) \rightarrow 0,\quad \theta (\xi ,\zeta ) \rightarrow 0, \quad \phi (\xi ,\zeta ) \rightarrow 0, \quad \chi (\xi ,\zeta ) \rightarrow 0\quad \text{ as } \zeta \rightarrow \infty . \end{aligned}$$

where, $\prime$ denotes the differentiation with respect to $\zeta$. Table 1 depicts the non-dimensional physical parameters introduced in the non-dimensional governing equations and Table 2 presents the physical properties of heat transfer fluid and suspended hybrid nanoparticles.

**Table 1:** Non-dimensional physical parameters influencing the fluid flow.

$A_1=\frac{\mu _{h n f}}{\mu _f}$	$A_2 = \frac{\rho _{h n f}}{\rho _f}$	$A_5=\frac{\left( \rho C_p\right) _{h n f}}{\left( \rho C_p\right) _f}$	$Pe= \frac{bW_c}{D_m}$
$A_3=\frac{\sigma _{hnf}}{\sigma _f}$	$A_4=\frac{k_{h n f}}{k_f}$	$N_\delta =\frac{N_\infty }{N_s-N_\infty }$	$Lb=\frac{\mu }{D_m}$
$M=\frac{\sigma B_{0}^{2}}{\rho _{f} b}$	$E_1=\frac{E_{0}}{B_{0} b(x+b)^{n}}$	$L = \frac{RD_{B}(N_{s}-N_{\infty })}{\kappa }$	$\delta _1 =\frac{C_{s}-C_{\infty }}{C_{\infty }}$
$L = \frac{RD_{B}(C_{s}-C_{\infty })}{\kappa }$	$Ec=\frac{U_{s}^{2}}{C_{p}\left( T_{s}-T_{\infty }\right) }$	$Pr=\frac{\mu C_{p}}{\kappa }$	$\delta =\frac{T_s-T_{\infty }}{T_{\infty }}$
$Rd=\frac{4\sigma ^* T^{3}_\infty }{\kappa _{f}\kappa ^*}$	$Nb=\frac{\rho c_{p} D_{B}\left( C_{s}-C_{\infty }\right) }{\rho _{f} C_{p} \nu }$	$Nt=\frac{\rho C_{p} D_{T}\left( T_{s}-T_{\infty }\right) }{\rho _{f} C_{p} \nu T_{\infty }}$	$Le = \frac{\nu }{D_{B}}$

**Table 2:** Physical properties of nanoparticles and basefluid (^62,63^).

Physical Properties	Silver	Graphene	Water
Density [$\rho (kg/m^3)$]	10, 500	2250	997
Heat Capacitance [$C_p(J/kgK)$]	235	2100	4179
Thermal Conductivity [$\kappa$(W/mK)]	429	2500	0.613
Electrical Conductivity [$\sigma$ (*S*/*m*)]	$6.3 \times 10^7$	$10^{7}$	0.05

### First-order truncation

In this step, the terms containing derivatives with respect to $\xi$ are neglected for $\xi< < 1$. Hence, after the first-order truncation equations from 3.1 to 3.4 are written as:

3.7 $$\begin{aligned} & \frac{\partial ^4 f}{\partial \zeta ^4}\bigg (\frac{n+1}{2}f+\frac{n-1}{2}\zeta \frac{\partial f}{\partial \zeta }\bigg )=\frac{2(1+\beta _1)}{A_1 \beta _2}\bigg [-\frac{A_1 K_p}{A_2 (n+1)} \frac{\partial f}{\partial \zeta }-\frac{Fr}{n+1} \bigg (\frac{\partial f}{\partial \zeta }\bigg )^2- \frac{A_3 M}{A_2 (n+1)} \bigg (\frac{\partial f}{\partial \zeta }-E_1\bigg )\bigg ]+\frac{1}{\beta _2}\frac{\partial ^3 f}{\partial \zeta ^3}\nonumber \\ & \quad + (n-1)\frac{\partial f}{\partial \zeta }\frac{\partial ^3 f}{\partial \zeta ^3}+n\bigg (\frac{\partial ^2 f}{\partial \zeta ^2}\bigg )^2-\frac{2(1+\beta _1)}{A_1 \beta _2}\bigg [\frac{n}{n+1}\bigg (\frac{\partial f}{\partial \zeta }\bigg )^2-\frac{f}{2}\frac{\partial ^2 f}{\partial \zeta ^2}-\frac{n-1}{2(n+1)} \zeta \frac{\partial ^2 f}{\partial \zeta ^2}\frac{\partial f}{\partial \zeta }\bigg ], \end{aligned}$$

3.8 $$\begin{aligned} & \quad \bigg (1+\frac{4}{3}Rd\bigg )\frac{\partial ^2 \theta }{\partial \zeta ^2}=- \frac{A_1 A_5}{A_2 A_4}\frac{\partial \theta }{\partial \zeta }\bigg (f+\frac{n-1}{n+1}\zeta \frac{\partial f}{\partial \zeta }\bigg )-\frac{A_3 A_1}{A_2 A_4}\frac{2}{n+1}Pr Ec M\bigg (\frac{\partial f}{\partial \zeta }-E_1\bigg )^2-\frac{A_5}{A_4}Pr Nb \frac{\partial \phi }{\partial \zeta }\frac{\partial \theta }{\partial \zeta }\nonumber \\ & \quad -\frac{A_5}{A_4}Pr Nt \bigg (\frac{\partial \theta }{\partial \zeta }\bigg )^2-\frac{A_1}{A_4}\frac{Pr Ec}{1+\beta _1}\bigg [\bigg (\frac{\partial ^2 f}{\partial \zeta ^2}\bigg )^2+\beta _2 \bigg [\bigg (\frac{\partial ^2 f}{\partial \zeta ^2}\bigg )^2\frac{\partial f}{\partial \zeta }-\frac{n+1}{2}\bigg (f+\frac{n-1}{n+1}\zeta \frac{\partial f}{\partial \zeta }\bigg )\frac{\partial ^2 f}{\partial \zeta ^2}\frac{\partial ^3 f}{\partial \zeta ^3} \bigg ]\bigg ],~~ \end{aligned}$$

3.9 $$\begin{aligned} & \quad \frac{\partial ^2 \phi }{\partial \zeta ^2}+\frac{Nt}{Nb}\frac{\partial ^2 \theta }{\partial \zeta ^2}=-\frac{A_1}{A_2}Le\bigg (f+\frac{n-1}{n+1}\zeta \frac{\partial f}{\partial \zeta }\bigg )\frac{\partial \phi }{\partial \zeta }, \end{aligned}$$

3.10 $$\begin{aligned} & \quad \frac{\partial ^2 \chi }{\partial \zeta ^2}=-\frac{A_1}{A_2}Lb\bigg (f+\frac{n-1}{n+1}\zeta \frac{\partial f}{\partial \zeta }\bigg ))\frac{\partial \chi }{\partial \zeta }+Pe\frac{\partial \phi }{\partial \zeta }\frac{\partial \chi }{\partial \zeta }+N_{\delta }\bigg (1+\frac{1}{N_{\delta }}\bigg )Pe \frac{\partial ^2 \phi }{\partial \zeta ^2}. \end{aligned}$$

Boundary conditions in dimensionless form:

3.11 $$\begin{aligned} & f(\xi , \zeta )+\frac{n-1}{n+1} \zeta f^\prime (\xi , \zeta )=0, \quad f^{\prime }(\xi ,\zeta )=1, \quad \theta (\xi ,\zeta )=1, \quad \phi (\xi ,\zeta )=1, \quad \chi (\xi ,\zeta )=1 \quad \text{ at } \quad \zeta =0, \end{aligned}$$

3.12 $$\begin{aligned} & \quad f^{\prime }(\xi ,\zeta ) \rightarrow 0,\quad \theta (\xi ,\zeta ) \rightarrow 0, \quad \phi (\xi ,\zeta ) \rightarrow 0, \quad \chi (\xi ,\zeta ) \rightarrow 0\quad \text{ as } \quad \zeta \rightarrow \infty . \end{aligned}$$

### Second-order truncation

In second-order truncation,

3.13 $$\begin{aligned} \frac{\partial f}{\partial \xi }=P(\xi ,\zeta ), \quad \frac{\partial \theta }{\partial \xi }=Q(\xi ,\zeta ), \quad \frac{\partial \phi }{\partial \xi }=R(\xi ,\zeta ), \quad \frac{\partial \chi }{\partial \xi }=S(\xi ,\zeta ). \end{aligned}$$

After introducing the equation 3.13 in equations 3.1 to 3.4, we get

3.14 $$\begin{aligned} & \frac{\partial ^4 f}{\partial \zeta ^4}\bigg (\xi P+\frac{n+1}{2}f+\frac{n-1}{2}\zeta \frac{\partial f}{\partial \zeta }\bigg )=\frac{2(1+\beta _1)}{A_1 \beta _2}\bigg [-\frac{A_1 K_p}{A_2 (n+1)} \frac{\partial f}{\partial \zeta }-\frac{Fr}{n+1} \bigg (\frac{\partial f}{\partial \zeta }\bigg )^2- \frac{A_3 M}{A_2 (n+1)} \bigg (\frac{\partial f}{\partial \zeta }-E_1\bigg )\bigg ]\nonumber \\ & \quad +\frac{1}{\beta _2}\frac{\partial ^3 f}{\partial \zeta ^3}+ (n-1)\frac{\partial f}{\partial \zeta }\frac{\partial ^3 f}{\partial \zeta ^3}+\xi \frac{\partial f}{\partial \zeta } \frac{\partial ^3 P}{\partial \zeta ^3}+n\bigg (\frac{\partial ^2 f}{\partial \zeta ^2}\bigg )^2+\xi \frac{\partial ^2 f}{\partial \zeta ^2} \frac{\partial ^2 P}{\partial \zeta ^2}-\xi \frac{\partial P}{\partial \zeta } \frac{\partial ^3 f}{\partial \zeta ^3}-\frac{2(1+\beta _1)}{A_1 \beta _2}\bigg [\frac{n}{n+1}\bigg (\frac{\partial f}{\partial \zeta }\bigg )^2 \nonumber \\ & \quad +\frac{\xi }{n+1}\frac{\partial f}{\partial \zeta } \frac{\partial P}{\partial \zeta }-\frac{\xi }{n+1}\frac{\partial ^2 f}{\partial \zeta ^2}P-\frac{f}{2}\frac{\partial ^2 f}{\partial \zeta ^2}-\frac{n-1}{2(n+1)} \zeta \frac{\partial ^2 f}{\partial \zeta ^2}\frac{\partial f}{\partial \zeta }\bigg ] \end{aligned}$$

3.15 $$\begin{aligned} & \quad \bigg (1+\frac{4}{3}Rd\bigg )\frac{\partial ^2 \theta }{\partial \zeta ^2}=\frac{2}{n+1}\frac{A_1 A_5}{A_2 A_4} \xi \frac{\partial f}{\partial \zeta }Q- \frac{A_1 A_5}{A_2 A_4}\frac{\partial \theta }{\partial \zeta }\bigg (\frac{2\xi }{n+1}P+f+\frac{n-1}{n+1}\zeta \frac{\partial f}{\partial \zeta }\bigg )-\frac{A_3 A_1}{A_2 A_4}\frac{2}{n+1}Pr Ec M\bigg (\frac{\partial f}{\partial \zeta }-E_1\bigg )^2\nonumber \\ & \quad -\frac{A_5}{A_4}Pr Nb \frac{\partial \phi }{\partial \zeta }\frac{\partial \theta }{\partial \zeta }-\frac{A_5}{A_4}Pr Nt \bigg (\frac{\partial \theta }{\partial \zeta }\bigg )^2-\frac{A_1}{A_4}\frac{Pr Ec}{1+\beta _1}\bigg [\bigg (\frac{\partial ^2 f}{\partial \zeta ^2}\bigg )^2+\beta _2 \bigg [\bigg (\frac{\partial ^2 f}{\partial \zeta ^2}\bigg )^2\frac{\partial f}{\partial \zeta }+\xi \frac{\partial ^2 P}{\partial \zeta ^2}\frac{\partial f}{\partial \zeta }\frac{\partial ^2 f}{\partial \zeta ^2}~~~\nonumber \\ & \quad -\frac{n+1}{2}\bigg (\frac{2\xi }{n+1} P+f+\frac{n-1}{n+1}\zeta \frac{\partial f}{\partial \zeta }\bigg )\frac{\partial ^2 f}{\partial \zeta ^2}\frac{\partial ^3 f}{\partial \zeta ^3} \bigg ]\bigg ],~~ \end{aligned}$$

3.16 $$\begin{aligned} & \quad \frac{\partial ^2 \phi }{\partial \zeta ^2}+\frac{Nt}{Nb}\frac{\partial ^2 \theta }{\partial \zeta ^2}=\frac{A_1}{A_2}\frac{2}{n+1}Le \xi \frac{\partial f}{\partial \zeta }R-\frac{A_1}{A_2}Le\bigg (\frac{2\xi }{n+1} P+f+\frac{n-1}{n+1}\zeta \frac{\partial f}{\partial \zeta }\bigg )\frac{\partial \phi }{\partial \zeta }, \end{aligned}$$

3.17 $$\begin{aligned} & \quad \frac{\partial ^2 \chi }{\partial \zeta ^2}=\frac{A_1}{A_2}\frac{2}{n+1}Lb \xi \frac{\partial f}{\partial \zeta }T-\frac{A_1}{A_2}Lb\bigg (\frac{2 \xi }{n+1}P+f+\frac{n-1}{n+1}\zeta \frac{\partial f}{\partial \zeta }\bigg )\frac{\partial R}{\partial \zeta }+Pe\frac{\partial \phi }{\partial \zeta }\frac{\partial \chi }{\partial \zeta }+N_{\delta }\bigg (1+\frac{1}{N_{\delta }}\bigg )Pe \frac{\partial ^2 \phi }{\partial \zeta ^2}. \end{aligned}$$

Boundary conditions:

3.18 $$\begin{aligned} & \frac{2 \xi }{n+1} P(\xi , \zeta )+f(\xi , \zeta )+\frac{n-1}{n+1} \zeta \frac{\partial f}{\partial \zeta }(\xi , \zeta )=0, \quad f^{\prime }(\xi ,\zeta )=1, \quad \theta (\xi ,\zeta )=1, \quad \phi (\xi ,\zeta )=1, \quad \chi (\xi ,\zeta )=1 \quad \text{ at } \zeta =0, \end{aligned}$$

3.19 $$\begin{aligned} & \quad f^{\prime }(\xi ,\zeta ) \rightarrow 0,\quad \theta (\xi ,\zeta ) \rightarrow 0, \quad \phi (\xi ,\zeta ) \rightarrow 0, \quad \chi (\xi ,\zeta ) \rightarrow 0\quad \text{ as } \zeta \rightarrow \infty . \end{aligned}$$

Here, *P*, *Q*, *R*, and *S* are the unknown functions in the equations 3.14-3.17. Hence, it is necessary to get the equations corresponding to these functions. For this purpose, the equations 3.14-3.17 are differentiated with respect to $\xi$.

3.20 $$\begin{aligned} & \frac{\partial ^4 f}{\partial \zeta ^4}\bigg (P+\frac{n+1}{2}P+\frac{n-1}{2}\zeta \frac{\partial P}{\partial \zeta }\bigg )+\frac{\partial ^4 P}{\partial \zeta ^4}\bigg (\xi P+\frac{n+1}{2}f+\frac{n-1}{2}\zeta \frac{\partial f}{\partial \zeta }\bigg )=\frac{2(1+\beta _1)}{A_1 \beta _2}\bigg [-\frac{A_1 K_p}{A_2 (n+1)} \frac{\partial P}{\partial \zeta }\nonumber \\ & \quad -\frac{Fr}{n+1}2 \bigg (\frac{\partial f}{\partial \zeta }\bigg )\bigg (\frac{\partial P}{\partial \zeta }\bigg )- \frac{A_3 M}{A_2 (n+1)} \bigg (\frac{\partial P}{\partial \zeta }\bigg )\bigg ]+\frac{1}{\beta _2}\frac{\partial ^3 P}{\partial \zeta ^3}+(n-1)\frac{\partial f}{\partial \zeta }\frac{\partial ^3 P}{\partial \zeta ^3}+(n-1)\frac{\partial P}{\partial \zeta }\frac{\partial ^3 f}{\partial \zeta ^3} + \frac{\partial f}{\partial \zeta } \frac{\partial ^3 P}{\partial \zeta ^3}\nonumber \\ & \quad +\xi \frac{\partial P}{\partial \zeta } \frac{\partial ^3 P}{\partial \zeta ^3} +2n\bigg (\frac{\partial ^2 f}{\partial \zeta ^2}\bigg )\bigg (\frac{\partial ^2 P}{\partial \zeta ^2}\bigg )+ \frac{\partial ^2 f}{\partial \zeta ^2} \frac{\partial ^2 P}{\partial \zeta ^2}+\xi \frac{\partial ^2 P}{\partial \zeta ^2} \frac{\partial ^2 P}{\partial \zeta ^2}- \frac{\partial P}{\partial \zeta } \frac{\partial ^3 f}{\partial \zeta ^3}-\xi \frac{\partial P}{\partial \zeta } \frac{\partial ^3 P}{\partial \zeta ^3} -\frac{2(1+\beta _1)}{A_1 \beta _2}\bigg [\frac{2n}{n+1} \nonumber \\ & \quad \bigg (\frac{\partial f}{\partial \zeta }\bigg )\bigg (\frac{\partial P}{\partial \zeta }\bigg )+\frac{1}{n+1}\frac{\partial f}{\partial \zeta } \frac{\partial P}{\partial \zeta }+\frac{\xi }{n+1}\frac{\partial P}{\partial \zeta } \frac{\partial P}{\partial \zeta }-\frac{1}{n+1}\frac{\partial ^2 f}{\partial \zeta ^2}P-\frac{\xi }{n+1}\frac{\partial ^2 P}{\partial \zeta ^2}P-\frac{P}{2}\frac{\partial ^2 f}{\partial \zeta ^2}-\frac{f}{2}\frac{\partial ^2 P}{\partial \zeta ^2} \nonumber \\ & \quad -\frac{n-1}{2(n+1)} \zeta \frac{\partial ^2 P}{\partial \zeta ^2}\frac{\partial f}{\partial \zeta }-\frac{n-1}{2(n+1)} \zeta \frac{\partial ^2 f}{\partial \zeta ^2}\frac{\partial P}{\partial \zeta }\bigg ] \end{aligned}$$

3.21 $$\begin{aligned} & \quad \bigg (1+\frac{4}{3}Rd\bigg )\frac{\partial ^2 Q}{\partial \zeta ^2}=\frac{2}{n+1}\frac{A_1 A_5}{A_2 A_4} \bigg (\frac{\partial f}{\partial \zeta }Q+\xi \frac{\partial P}{\partial \zeta }Q\bigg )- \frac{A_1 A_5}{A_2 A_4}\bigg (\frac{\partial \theta }{\partial \zeta }\bigg (\frac{2}{n+1}P+P+\frac{n-1}{n+1}\zeta \frac{\partial P}{\partial \zeta }\bigg )+\frac{\partial Q}{\partial \zeta }\nonumber \\ & \quad \bigg (\frac{2\xi }{n+1}P+f+\frac{n-1}{n+1}\zeta \frac{\partial f}{\partial \zeta }\bigg )\bigg )-\frac{A_3 A_1}{A_2 A_4}\frac{2}{n+1}Pr Ec M 2\bigg (\frac{\partial f}{\partial \zeta }-E_1\bigg )\bigg (\frac{\partial P}{\partial \zeta }\bigg )-\frac{A_5}{A_4}Pr Nb \bigg (\frac{\partial \phi }{\partial \zeta }\frac{\partial Q}{\partial \zeta }+\frac{\partial R}{\partial \zeta }\frac{\partial \theta }{\partial \zeta }\bigg )\nonumber \\ & \quad -\frac{2A_5}{A_4}Pr Nt\bigg (\frac{\partial \theta }{\partial \zeta }\bigg )\bigg (\frac{\partial Q}{\partial \zeta }\bigg )-\frac{2A_1}{A_4}\frac{Pr Ec}{1+\beta _1}\bigg [\bigg (\frac{\partial ^2 f}{\partial \zeta ^2}\bigg )\bigg (\frac{\partial ^2 P}{\partial \zeta ^2}\bigg )+\beta _2 \bigg [ \bigg (\frac{\partial ^2 f}{\partial \zeta ^2}\bigg )^2\frac{\partial P}{\partial \zeta }+2\bigg (\frac{\partial ^2 f}{\partial \zeta ^2}\bigg )\bigg (\frac{\partial ^2 P}{\partial \zeta ^2}\bigg )\frac{\partial f}{\partial \zeta } +\xi \frac{\partial ^2 P}{\partial \zeta ^2}\nonumber \\ & \quad \frac{\partial P}{\partial \zeta }\frac{\partial ^2 f}{\partial \zeta ^2}+\xi \frac{\partial ^2 P}{\partial \zeta ^2}\frac{\partial f}{\partial \zeta }\frac{\partial ^2 P}{\partial \zeta ^2}+\frac{\partial ^2 P}{\partial \zeta ^2}\frac{\partial f}{\partial \zeta }\frac{\partial ^2 f}{\partial \zeta ^2}-\frac{n+1}{2}\bigg [\bigg (\frac{2\xi }{n+1} P+f+\frac{n-1}{n+1}\zeta \frac{\partial f}{\partial \zeta }\bigg )\bigg (\frac{\partial ^2 f}{\partial \zeta ^2}\frac{\partial ^3 P}{\partial \zeta ^3}+\frac{\partial ^2 P}{\partial \zeta ^2}\frac{\partial ^3 f}{\partial \zeta ^3}\bigg )\nonumber \\ & \quad +\bigg (\frac{2}{n+1} P+P+\frac{n-1}{n+1}\zeta \frac{\partial P}{\partial \zeta }\bigg )\frac{\partial ^2 f}{\partial \zeta ^2}\frac{\partial ^3 f}{\partial \zeta ^3} \bigg ]\bigg ]\bigg ], \end{aligned}$$

3.22 $$\begin{aligned} & \quad \frac{\partial ^2 R}{\partial \zeta ^2}=\frac{A_1}{A_2}\frac{2}{n+1}Le R\bigg (\xi \frac{\partial P}{\partial \zeta }+ \frac{\partial f}{\partial \zeta }\bigg )-\frac{A_1}{A_2}Le\bigg [\bigg (\frac{2 \xi }{n+1} P+f+\frac{n-1}{n+1}\zeta \frac{\partial f}{\partial \zeta }\bigg )\frac{\partial R}{\partial \zeta }+\bigg (\frac{2}{n+1} P+P+\frac{n-1}{n+1}\zeta \frac{\partial P}{\partial \zeta }\bigg )\frac{\partial \phi }{\partial \zeta }\bigg ]\nonumber \\ & \quad -\frac{Nt}{Nb}\frac{\partial ^2 Q}{\partial \zeta ^2}, \end{aligned}$$

3.23 $$\begin{aligned} & \quad \frac{\partial ^2 S}{\partial \zeta ^2}=\frac{A_1}{A_2}\frac{2}{n+1}LbS\bigg (\xi \frac{\partial P}{\partial \zeta }+\frac{\partial f}{\partial \zeta }\bigg ) -\frac{A_1}{A_2}Lb\bigg [\bigg (\frac{2 }{n+1}P+P+\frac{n-1}{n+1}\zeta \frac{\partial P}{\partial \zeta }\bigg )\frac{\partial \chi }{\partial \zeta }+\bigg (\frac{2 \xi }{n+1}P+f+\frac{n-1}{n+1}\zeta \frac{\partial f}{\partial \zeta }\bigg )\frac{\partial S}{\partial \zeta }\bigg ] \nonumber \\ & \quad +Pe\bigg (\frac{\partial \phi }{\partial \zeta }\frac{\partial S}{\partial \zeta }+\frac{\partial R}{\partial \zeta }\frac{\partial \chi }{\partial \zeta }\bigg )+N_{\delta }\bigg (1+\frac{1}{N_{\delta }}\bigg )Pe \frac{\partial ^2 R}{\partial \zeta ^2}. \end{aligned}$$

Boundary conditions:

3.24 $$\begin{aligned} & \frac{2}{n+1} P(\xi , \zeta )+P(\xi , \zeta )+\frac{n-1}{n+1} \zeta P^\prime (\xi , \zeta )=0, \quad P^{\prime }(\xi ,\zeta )=0, \quad Q(\xi ,\zeta )=0, \quad R(\xi ,\zeta )=0, \quad S(\xi ,\zeta )=0 \quad \text{ at } \zeta =0, \end{aligned}$$

3.25 $$\begin{aligned} & \quad P^{\prime }(\xi ,\zeta ) \rightarrow 0,\quad Q(\xi ,\zeta ) \rightarrow 0, \quad R(\xi ,\zeta ) \rightarrow 0, \quad S(\xi ,\zeta ) \rightarrow 0\quad \text{ as } \zeta \rightarrow \infty . \end{aligned}$$

In the third level truncation, the terms containing $\frac{\partial P}{\partial \xi }$, $\frac{\partial Q}{\partial \xi }$, $\frac{\partial R}{\partial \xi }$, and $\frac{\partial T}{\partial \xi }$ are truncated. But in the equations from 3.20 to 3.25, there is no term of *P* , *Q*, *R*, and *S* containing derivatives with respect to $\xi$. Hence, the equations from 3.20 to 3.25 remain unchanged after the second-order truncation; therefore, the non-similar solution up to the second-order truncation give accurate simulations.

## Cash and Carp methodology

This section employs advanced numerical techniques known as Cash and Carp method to solve coupled ordinary differential equations (ODEs) by changing them into system of first-order ODEs. The system of ODEs has eight functions namely *f*, *P*, $\theta$, *Q*, $\phi$, *S*, $\chi$, and *S*. Now, the ODEs for the functions *f*, *P*, $\theta$, *Q*, $\phi$, *S*, $\chi$, and *S* and their boundary conditions are collected by assuming $\xi$ as parameter as given below :

4.1 $$\begin{aligned} & \frac{\partial ^4 f}{\partial \zeta ^4}\bigg (\xi P+\frac{n+1}{2}f+\frac{n-1}{2}\zeta \frac{\partial f}{\partial \zeta }\bigg )=\frac{2(1+\beta _1)}{A_1 \beta _2}\bigg [-\frac{A_1 K_p}{A_2 (n+1)} \frac{\partial f}{\partial \zeta }-\frac{Fr}{n+1} \bigg (\frac{\partial f}{\partial \zeta }\bigg )^2- \frac{A_3 M}{A_2 (n+1)} \bigg (\frac{\partial f}{\partial \zeta }-E_1\bigg )\bigg ]\nonumber \\ & \quad +\frac{1}{\beta _2}\frac{\partial ^3 f}{\partial \zeta ^3}+ (n-1)\frac{\partial f}{\partial \zeta }\frac{\partial ^3 f}{\partial \zeta ^3}+\xi \frac{\partial f}{\partial \zeta } \frac{\partial ^3 P}{\partial \zeta ^3}+n\bigg (\frac{\partial ^2 f}{\partial \zeta ^2}\bigg )^2+\xi \frac{\partial ^2 f}{\partial \zeta ^2} \frac{\partial ^2 P}{\partial \zeta ^2}-\xi \frac{\partial P}{\partial \zeta } \frac{\partial ^3 f}{\partial \zeta ^3}-\frac{2(1+\beta _1)}{A_1 \beta _2}\bigg [\frac{n}{n+1}\bigg (\frac{\partial f}{\partial \zeta }\bigg )^2]\nonumber \\ & \quad +\frac{\xi }{n+1}\frac{\partial f}{\partial \zeta } \frac{\partial P}{\partial \zeta }-\frac{\xi }{n+1}\frac{\partial ^2 f}{\partial \zeta ^2}P-\frac{f}{2}\frac{\partial ^2 f}{\partial \zeta ^2}-\frac{n-1}{2(n+1)} \zeta \frac{\partial ^2 f}{\partial \zeta ^2}\frac{\partial f}{\partial \zeta }\bigg ] \end{aligned}$$

4.2 $$\begin{aligned} & \quad \frac{\partial ^4 f}{\partial \zeta ^4}\bigg (P+\frac{n+1}{2}P+\frac{n-1}{2}\zeta \frac{\partial P}{\partial \zeta }\bigg )+\frac{\partial ^4 P}{\partial \zeta ^4}\bigg (\xi P+\frac{n+1}{2}f+\frac{n-1}{2}\zeta \frac{\partial f}{\partial \zeta }\bigg )=\frac{2(1+\beta _1)}{A_1 \beta _2}\bigg [-\frac{A_1 K_p}{A_2 (n+1)} \frac{\partial P}{\partial \zeta }]\nonumber \\ & \quad -\frac{Fr}{n+1}2 \bigg (\frac{\partial f}{\partial \zeta }\bigg )\bigg (\frac{\partial P}{\partial \zeta }\bigg )- \frac{A_3 M}{A_2 (n+1)} \bigg (\frac{\partial P}{\partial \zeta }\bigg )\bigg ]+\frac{1}{\beta _2}\frac{\partial ^3 P}{\partial \zeta ^3}+(n-1)\frac{\partial f}{\partial \zeta }\frac{\partial ^3 P}{\partial \zeta ^3}+(n-1)\frac{\partial P}{\partial \zeta }\frac{\partial ^3 f}{\partial \zeta ^3} + \frac{\partial f}{\partial \zeta } \frac{\partial ^3 P}{\partial \zeta ^3}]\nonumber \\ & \quad +\xi \frac{\partial P}{\partial \zeta } \frac{\partial ^3 P}{\partial \zeta ^3} +2n\bigg (\frac{\partial ^2 f}{\partial \zeta ^2}\bigg )\bigg (\frac{\partial ^2 P}{\partial \zeta ^2}\bigg )+ \frac{\partial ^2 f}{\partial \zeta ^2} \frac{\partial ^2 P}{\partial \zeta ^2}+\xi \frac{\partial ^2 P}{\partial \zeta ^2} \frac{\partial ^2 P}{\partial \zeta ^2}- \frac{\partial P}{\partial \zeta } \frac{\partial ^3 f}{\partial \zeta ^3}-\xi \frac{\partial P}{\partial \zeta } \frac{\partial ^3 P}{\partial \zeta ^3} -\frac{2(1+\beta _1)}{A_1 \beta _2}\bigg [\frac{2n}{n+1} ]\nonumber \\ & \quad \bigg (\frac{\partial f}{\partial \zeta }\bigg )\bigg (\frac{\partial P}{\partial \zeta }\bigg )+\frac{1}{n+1}\frac{\partial f}{\partial \zeta } \frac{\partial P}{\partial \zeta }+\frac{\xi }{n+1}\frac{\partial P}{\partial \zeta } \frac{\partial P}{\partial \zeta }-\frac{1}{n+1}\frac{\partial ^2 f}{\partial \zeta ^2}P-\frac{\xi }{n+1}\frac{\partial ^2 P}{\partial \zeta ^2}P-\frac{P}{2}\frac{\partial ^2 f}{\partial \zeta ^2}-\frac{f}{2}\frac{\partial ^2 P}{\partial \zeta ^2} ]\nonumber \\ & \quad -\frac{n-1}{2(n+1)} \zeta \frac{\partial ^2 P}{\partial \zeta ^2}\frac{\partial f}{\partial \zeta }-\frac{n-1}{2(n+1)} \zeta \frac{\partial ^2 f}{\partial \zeta ^2}\frac{\partial P}{\partial \zeta }\bigg ] \end{aligned}$$

4.3 $$\begin{aligned} & \quad \bigg (1+\frac{4}{3}Rd\bigg )\frac{\partial ^2 \theta }{\partial \zeta ^2}=\frac{2}{n+1}\frac{A_1 A_5}{A_2 A_4} \xi \frac{\partial f}{\partial \zeta }Q- \frac{A_1 A_5}{A_2 A_4}\frac{\partial \theta }{\partial \zeta }\bigg (\frac{2\xi }{n+1}P+f+\frac{n-1}{n+1}\zeta \frac{\partial f}{\partial \zeta }\bigg )-\frac{A_3 A_1}{A_2 A_4}\frac{2}{n+1}Pr Ec M\bigg (\frac{\partial f}{\partial \zeta }-E_1\bigg )^2]\nonumber \\ & \quad -\frac{A_5}{A_4}Pr Nb \frac{\partial \phi }{\partial \zeta }\frac{\partial \theta }{\partial \zeta }-\frac{A_5}{A_4}Pr Nt \bigg (\frac{\partial \theta }{\partial \zeta }\bigg )^2-\frac{A_1}{A_4}\frac{Pr Ec}{1+\beta _1}\bigg [\bigg (\frac{\partial ^2 f}{\partial \zeta ^2}\bigg )^2+\beta _2 \bigg [\bigg (\frac{\partial ^2 f}{\partial \zeta ^2}\bigg )^2\frac{\partial f}{\partial \zeta }+\xi \frac{\partial ^2 P}{\partial \zeta ^2}\frac{\partial f}{\partial \zeta }\frac{\partial ^2 f}{\partial \zeta ^2}~~~]\nonumber \\ & \quad -\frac{n+1}{2}\bigg (\frac{2\xi }{n+1} P+f+\frac{n-1}{n+1}\zeta \frac{\partial f}{\partial \zeta }\bigg )\frac{\partial ^2 f}{\partial \zeta ^2}\frac{\partial ^3 f}{\partial \zeta ^3} \bigg ]\bigg ],~~ \end{aligned}$$

4.4 $$\begin{aligned} & \quad \bigg (1+\frac{4}{3}Rd\bigg )\frac{\partial ^2 Q}{\partial \zeta ^2}=\frac{2}{n+1}\frac{A_1 A_5}{A_2 A_4} \bigg (\frac{\partial f}{\partial \zeta }Q+\xi \frac{\partial P}{\partial \zeta }Q\bigg )- \frac{A_1 A_5}{A_2 A_4}\bigg (\frac{\partial \theta }{\partial \zeta }\bigg (\frac{2}{n+1}P+P+\frac{n-1}{n+1}\zeta \frac{\partial P}{\partial \zeta }\bigg )+\frac{\partial Q}{\partial \zeta }]\nonumber \\ & \quad \bigg (\frac{2\xi }{n+1}P+f+\frac{n-1}{n+1}\zeta \frac{\partial f}{\partial \zeta }\bigg )\bigg )-\frac{A_3 A_1}{A_2 A_4}\frac{2}{n+1}Pr Ec M 2\bigg (\frac{\partial f}{\partial \zeta }-E_1\bigg )\bigg (\frac{\partial P}{\partial \zeta }\bigg )-\frac{A_5}{A_4}Pr Nb \bigg (\frac{\partial \phi }{\partial \zeta }\frac{\partial R}{\partial \zeta }+\frac{\partial R}{\partial \zeta }\frac{\partial \theta }{\partial \zeta }\bigg )]\nonumber \\ & \quad -\frac{2A_5}{A_4}Pr Nt\bigg (\frac{\partial \theta }{\partial \zeta }\bigg )\bigg (\frac{\partial Q}{\partial \zeta }\bigg )-\frac{2A_1}{A_4}\frac{Pr Ec}{1+\beta _1}\bigg [\bigg (\frac{\partial ^2 f}{\partial \zeta ^2}\bigg )\bigg (\frac{\partial ^2 P}{\partial \zeta ^2}\bigg )+\beta _2 \bigg [ \bigg (\frac{\partial ^2 f}{\partial \zeta ^2}\bigg )^2\frac{\partial P}{\partial \zeta }+2\bigg (\frac{\partial ^2 f}{\partial \zeta ^2}\bigg )\bigg (\frac{\partial ^2 P}{\partial \zeta ^2}\bigg )\frac{\partial f}{\partial \zeta } +\xi \frac{\partial ^2 P}{\partial \zeta ^2}]\nonumber \\ & \quad \frac{\partial P}{\partial \zeta }\frac{\partial ^2 f}{\partial \zeta ^2}+\xi \frac{\partial ^2 P}{\partial \zeta ^2}\frac{\partial f}{\partial \zeta }\frac{\partial ^2 P}{\partial \zeta ^2}+\frac{\partial ^2 P}{\partial \zeta ^2}\frac{\partial f}{\partial \zeta }\frac{\partial ^2 f}{\partial \zeta ^2}-\frac{n+1}{2}\bigg [\bigg (\frac{2\xi }{n+1} P+f+\frac{n-1}{n+1}\zeta \frac{\partial f}{\partial \zeta }\bigg )\bigg (\frac{\partial ^2 f}{\partial \zeta ^2}\frac{\partial ^3 P}{\partial \zeta ^3}+\frac{\partial ^2 P}{\partial \zeta ^2}\frac{\partial ^3 f}{\partial \zeta ^3}\bigg )]\nonumber \\ & \quad +\bigg (\frac{2}{n+1} P+P+\frac{n-1}{n+1}\zeta \frac{\partial P}{\partial \zeta }\bigg )\frac{\partial ^2 f}{\partial \zeta ^2}\frac{\partial ^3 f}{\partial \zeta ^3} \bigg ]\bigg ]\bigg ], \end{aligned}$$

4.5 $$\begin{aligned} & \quad \frac{\partial ^2 \phi }{\partial \zeta ^2}+\frac{Nt}{Nb}\frac{\partial ^2 \theta }{\partial \zeta ^2}=\frac{A_1}{A_2}\frac{2}{n+1}Le \xi \frac{\partial f}{\partial \zeta }R-\frac{A_1}{A_2}Le\bigg (\frac{2\xi }{n+1} P+f+\frac{n-1}{n+1}\zeta \frac{\partial f}{\partial \zeta }\bigg )\frac{\partial \phi }{\partial \zeta }, \end{aligned}$$

4.6 $$\begin{aligned} & \quad \frac{\partial ^2 R}{\partial \zeta ^2}=\frac{A_1}{A_2}\frac{2}{n+1}Le R\bigg (\xi \frac{\partial P}{\partial \zeta }+ \frac{\partial f}{\partial \zeta }\bigg )-\frac{A_1}{A_2}Le\bigg [\bigg (\frac{2 \xi }{n+1} P+f+\frac{n-1}{n+1}\zeta \frac{\partial f}{\partial \zeta }\bigg )\frac{\partial R}{\partial \zeta }+\bigg (\frac{2}{n+1} P+P+\frac{n-1}{n+1}\zeta \frac{\partial P}{\partial \zeta }\bigg )\frac{\partial \phi }{\partial \zeta }\bigg ]]\nonumber \\ & \quad -\frac{Nt}{Nb}\frac{\partial ^2 Q}{\partial \zeta ^2},~~~ \end{aligned}$$

4.7 $$\begin{aligned} & \quad \frac{\partial ^2 \chi }{\partial \zeta ^2}=\frac{A_1}{A_2}\frac{2}{n+1}Lb \xi \frac{\partial f}{\partial \zeta }T-\frac{A_1}{A_2}Lb\bigg (\frac{2 \xi }{n+1}P+f+\frac{n-1}{n+1}\zeta \frac{\partial f}{\partial \zeta }\bigg )\frac{\partial R}{\partial \zeta }+Pe\frac{\partial \phi }{\partial \zeta }\frac{\partial \chi }{\partial \zeta }+N_{\delta }\bigg (1+\frac{1}{N_{\delta }}\bigg )Pe \frac{\partial ^2 \phi }{\partial \zeta ^2}. \end{aligned}$$

4.8 $$\begin{aligned} & \quad \frac{\partial ^2 S}{\partial \zeta ^2}=\frac{A_1}{A_2}\frac{2}{n+1}LbS\bigg (\xi \frac{\partial P}{\partial \zeta }+\frac{\partial f}{\partial \zeta }\bigg ) -\frac{A_1}{A_2}Lb\bigg [\bigg (\frac{2 }{n+1}P+P+\frac{n-1}{n+1}\zeta \frac{\partial P}{\partial \zeta }\bigg )\frac{\partial \chi }{\partial \zeta }+\bigg (\frac{2 \xi }{n+1}P+f+\frac{n-1}{n+1}\zeta \frac{\partial f}{\partial \zeta }\bigg )\frac{\partial S}{\partial \zeta }\bigg ]]\nonumber \\ & \quad +Pe\bigg (\frac{\partial \phi }{\partial \zeta }\frac{\partial S}{\partial \zeta }+\frac{\partial R}{\partial \zeta }\frac{\partial \chi }{\partial \zeta }\bigg )+N_{\delta }\bigg (1+\frac{1}{N_{\delta }}\bigg )Pe \frac{\partial ^2 R}{\partial \zeta ^2}.~~~ \end{aligned}$$

Boundary conditions:

4.9 $$\begin{aligned} & \frac{2 \xi }{n+1} P(\xi , \zeta )+f(\xi , \zeta )+\frac{n-1}{n+1} \zeta f^\prime (\xi , \zeta )=0, \quad f^{\prime }(\xi ,\zeta )=1, \quad \frac{2}{n+1} P(\xi , \zeta )+P(\xi , \zeta )+\frac{n-1}{n+1} \zeta P^\prime (\xi , \zeta )=0, \quad ]\nonumber \\ & \quad P^{\prime }(\xi ,\zeta )=0, \quad \theta (\xi ,\zeta )=1, \quad Q(\xi ,\zeta )=0, \quad \phi (\xi ,\zeta )=1, \quad R(\xi ,\zeta )=0, \quad \chi (\xi ,\zeta )=1 \quad S(\xi ,\zeta )=0 \quad \text{ at } \zeta =0, \end{aligned}$$

4.10 $$\begin{aligned} & \quad f^{\prime }(\xi ,\zeta ) \rightarrow 0, \quad P^{\prime }(\xi ,\zeta ) \rightarrow 0, \quad \theta (\xi ,\zeta ) \rightarrow 0, \quad Q(\xi ,\zeta ) \rightarrow 0, \quad \phi (\xi ,\zeta ) \rightarrow 0, \quad R(\xi ,\zeta ) \rightarrow 0, \quad \chi (\xi ,\zeta ) \rightarrow 0]\nonumber \\ & \quad \quad S(\xi ,\zeta ) \rightarrow 0\quad \text{ as } \zeta \rightarrow \infty . \end{aligned}$$

System of first-order ODEs along with boundary conditions is made by making the following assumptions like,

4.11 $$\begin{aligned} {\left\{ \begin{array}{ll} f=f(1),\quad f^\prime =f(2), \quad f^{\prime \prime }=f(3), \quad f^{\prime \prime \prime }=f(4), \\ P=f(5),\quad P^\prime =f(6), \quad P^{\prime \prime }=f(7), \quad P^{\prime \prime \prime }=f(8), \quad \\ \theta =f(9), \quad \theta ^\prime =f(10), \\ Q=f(11),\quad Q^\prime =f(12), \\ \phi =f(13), \quad \phi ^\prime =f(14), \\ R=f(15), \quad R^\prime =f(16), \\ \chi =f(17), \quad \chi ^\prime =f(18),\\ S=f(19), \quad S^\prime =f(20), \end{array}\right. } \end{aligned}$$

**Fig. 2:**
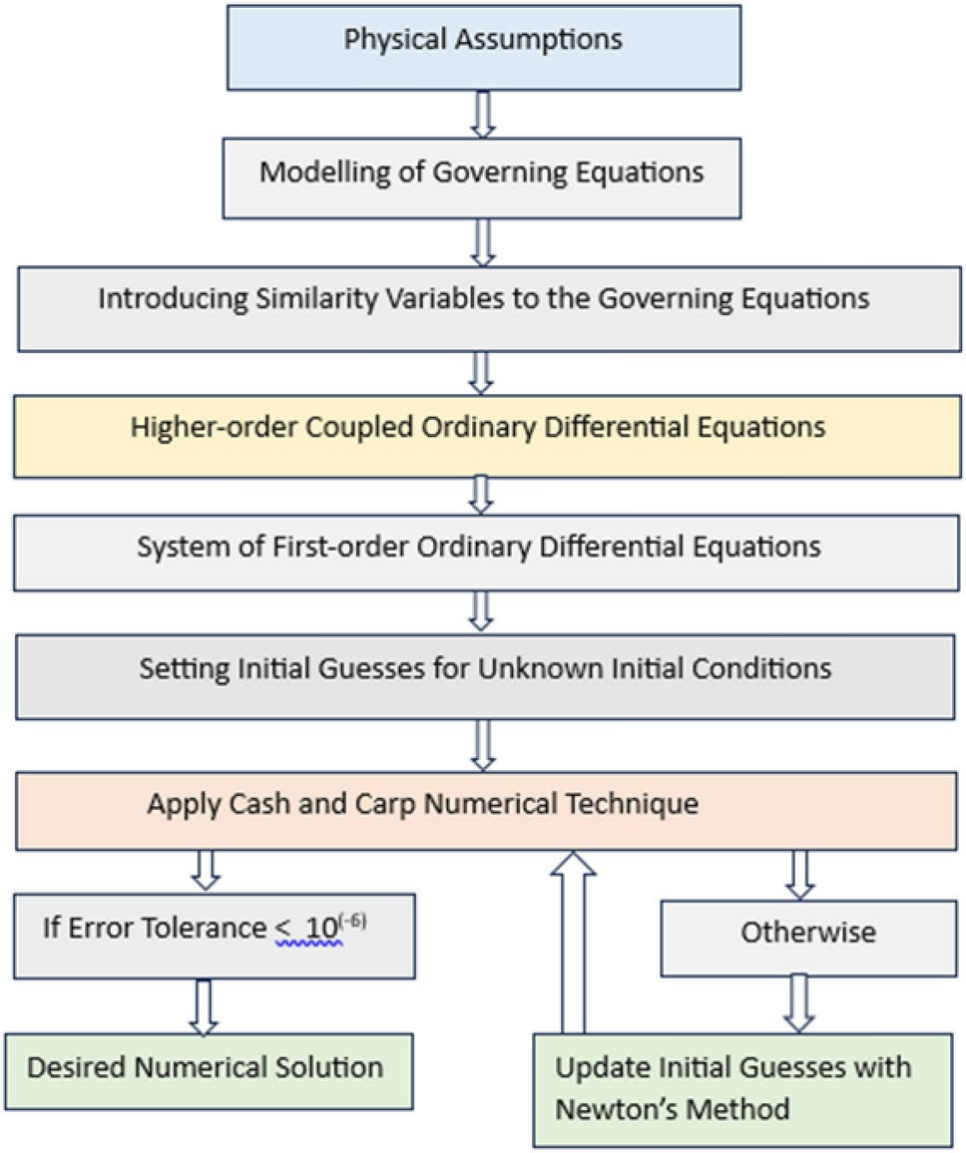
Flowchart presenting numerical methodology.

Now, Cash and Carp methodology has been applied to solve the system of ODEs. Cash and Carp method necessitates four initial conditions for the momentum equation and two for the energy, concentration, and microorganism concentration equation. However, the available initial conditions consist of only two for the momentum equation and one each for the energy, concentration, and microorganism dispersion equations. The selection of the step size h is a critical consideration in problem-solving. In the context of this problem, the value of h is defined as 0.001. System of equations analyzed numerically using the fifth-order approach. The numerical solution is updated continuously until the error tolerance becomes less than $10^{-6}$. A schematic flow chart outlining the details of numerical method employed in this analysis is depicted in Fig. 2 (Table 3).

**Table 3:** The indices representations for Cash and Carp methodology.

i	$A_1$			$B_{i j}$			$C_i$	$D_i$
1	–	–	–	–	–	–	37/378	2825/27648
2	1/5	1/5	–	–	–	–	0	0
3	3/10	3/40	9/40	–	–	–	250/621	18575/48384
4	3/5	3/10	9/40	6/5	–	–	125/594	13525/55296
5	1	11/54	5/2	70/27	35/27	–	0	277/14336
6	7/8	1631/55296	175/512	575/13824	44275/110592	253/4096	512/1771	1/4

The expression for Cash and Carp numerical method is written as:

$$\begin{aligned} \left. \begin{array}{l} \textbf{K}_{\textbf{1}}=h \textbf{F}(\zeta , \textbf{Z}), \\ \textbf{K}_{\textbf{i}}=h \textbf{F}\left( \zeta +A_{\textbf{i}}, \textbf{Z}+\sum _{j=1}^{i-1} B_{i j} \textbf{K}_{\textbf{j}}\right) , i=2,3, \ldots , 6, \\ \textbf{Z}_5(\zeta +h)=\textbf{Z}(\zeta )+\sum _{i=1}^6 C_{\textbf{i}} \textbf{K}_{\textbf{i}}, \\ \textbf{Z}_4(\zeta +h)=\textbf{Z}(\zeta )+\sum _{i=1}^6 D_i \textbf{K}_{\textbf{i}}, \end{array}\right\} \end{aligned}$$

However, the fourth-order technique is employed due to specific reasons related to truncation error.

$$\begin{aligned} E(h)=Z_5(\zeta +h)-Z_4(\zeta +h)=\sum _i\left( C_i-D_i\right) K_i \end{aligned}$$

The difference between numerically determined numbers and boundary values is referred to as the “residual”. The final solution has been reached if the residuals at the boundary conditions is less than the error tolerance of $10^{-6}$. Newton’s iterative technique is applied and the following standard decided the finishing of iterative process:

$$\begin{aligned} \max \left\{ \left| z\left( \zeta _{\max }\right) -z(\zeta _{end})\right| \right\} <10^{-6} \end{aligned}$$

where, $z\left( \zeta _{\max }\right)$ is the calculated output from the numerical scheme and $z(\zeta _{end})$ is the given boundary condition for the respective function.

## Artificial neural network modeling

**Fig. 3:**
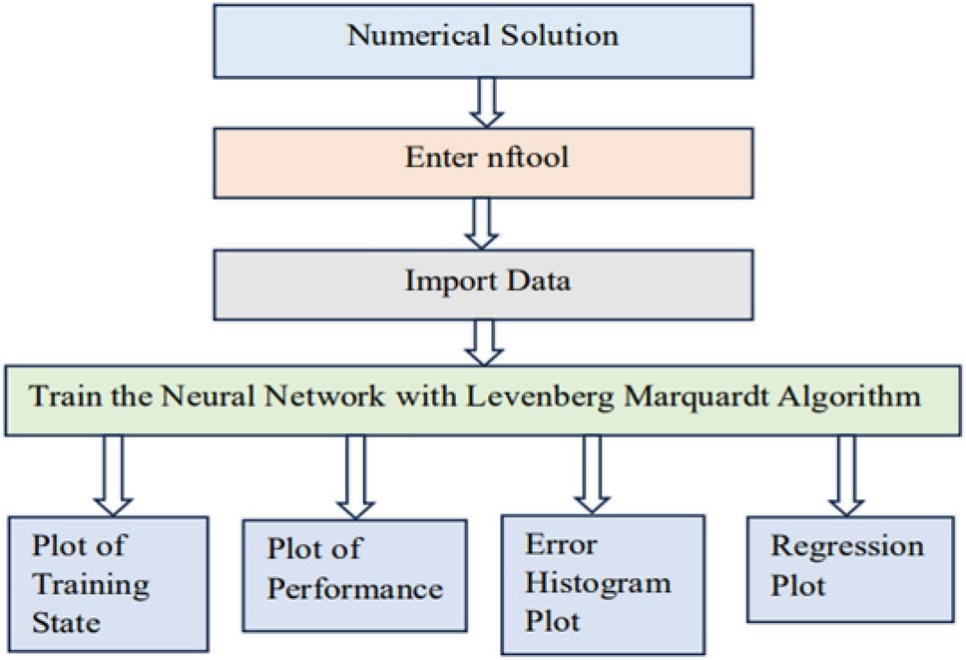
An illustration of the mathematical model developed for the artificial neural network.

**Fig. 4:**
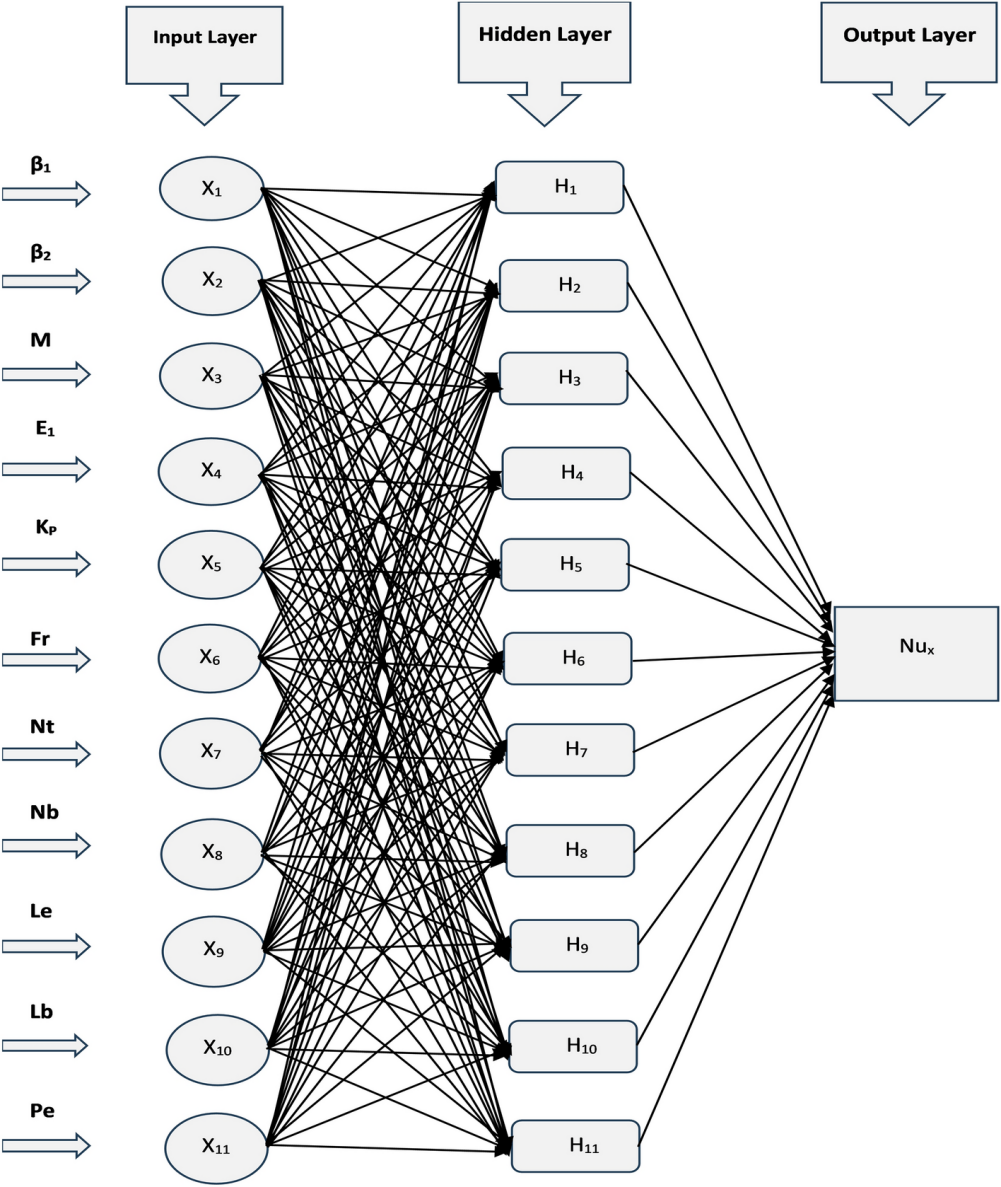
The basic configuration architecture of the developed artificial neural network.

**Fig. 5:**
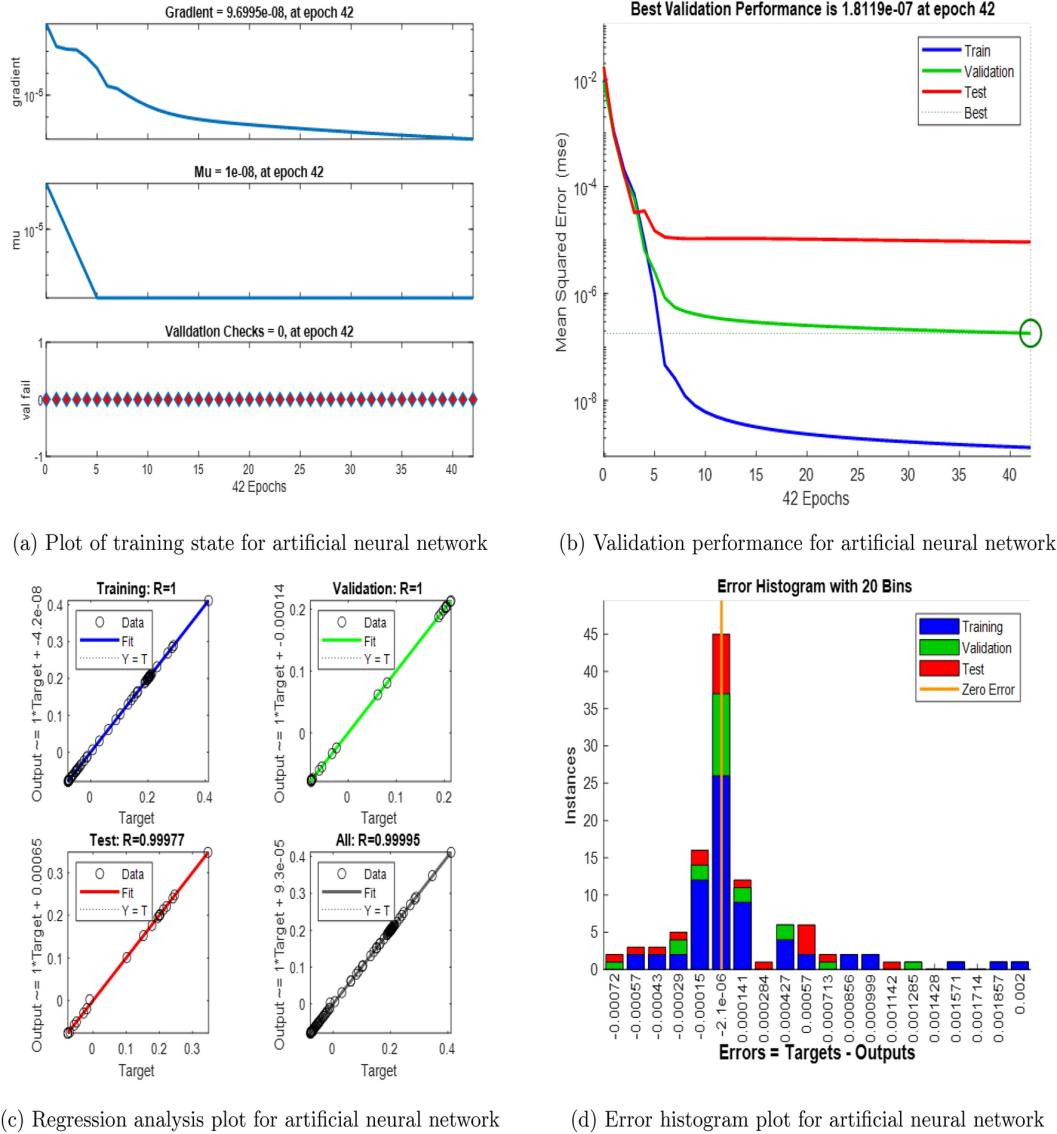
Outputs of the trained artificial neural network.

An artificial neural network emulates biological neural networks found in the brain and is designed for computational purposes. It comprises interconnected nodes known as neurons, structured into layers including input, hidden, and output layers. The input layer provides data where each node represents a specific feature. This data processed by the hidden layers using activation functions and weight connections. Neurons in these layers compute inputs, apply weights, sum them, and pass through activation functions to introduce non-linear transformations. The output layer generates final predictions or classifications based on the processed data. Training an artificial neural network involves optimizing the connection weights between neurons to diminish the difference of the predicted and actual outputs. This can be done by algorithms of optimization like gradient descent and backpropagation. The artificial neural network is developed for predictions of the Nusselt number with corresponding values of different influential physical parameters such as Deborah number ($\beta _1$), retardation time parameter ($\beta _2$), magnetic field parameter (*M*), electric field parameter ($E_1$), Darcy number ($K_p$), Forchheimer number (*Fr*), thermophoretic diffusion parameter (*Nt*), Brownian motion parameter (*Nb*), Lewis number (*Le*), bioconvection Lewis number (*Lb*), and Peclet number (*Pe*). 10 default values for each physical parameter are taken to address the neural network having values varies from the 0.1 to 1 with time step 0.1. The “nftool” is selected in the Matlab Computational Software to train the neural network. Input and output data are than imported to the “nftool”. The imported data is then divided into two parts, in which 70% of the data is taken to train the neural network and 30% for validation and test purposes. The data is trained in the “nftool” with the Levenberg Marquardt algorithm. The flowchart and the basic configuration architecture of the designed neural network is shown in Figs. 3 and 4. And the corresponding numerical results are presented in the Fig. 5.

Artificial neural network outcomes for the trained network are shown in the Figs. 5a–d. Figure 5a presents the plot of training state for developed artificial neural network and the training completed in the 42 epoch with gradient value of $9.6995e-08$ and Mu value $1e-08$. Figure 5b presents the plot of validation performance for the trained neural network. In this Figure, the best validation performance is $1.8119e-07$ and occur at 42 epoch. Figure 5c shows the plot of regression analysis for the training, validation and test performance. Figure 5d depicts the error histogram plot for trained network corresponding to the training, validation and test.

## Results and discussion

**Fig. 6:**
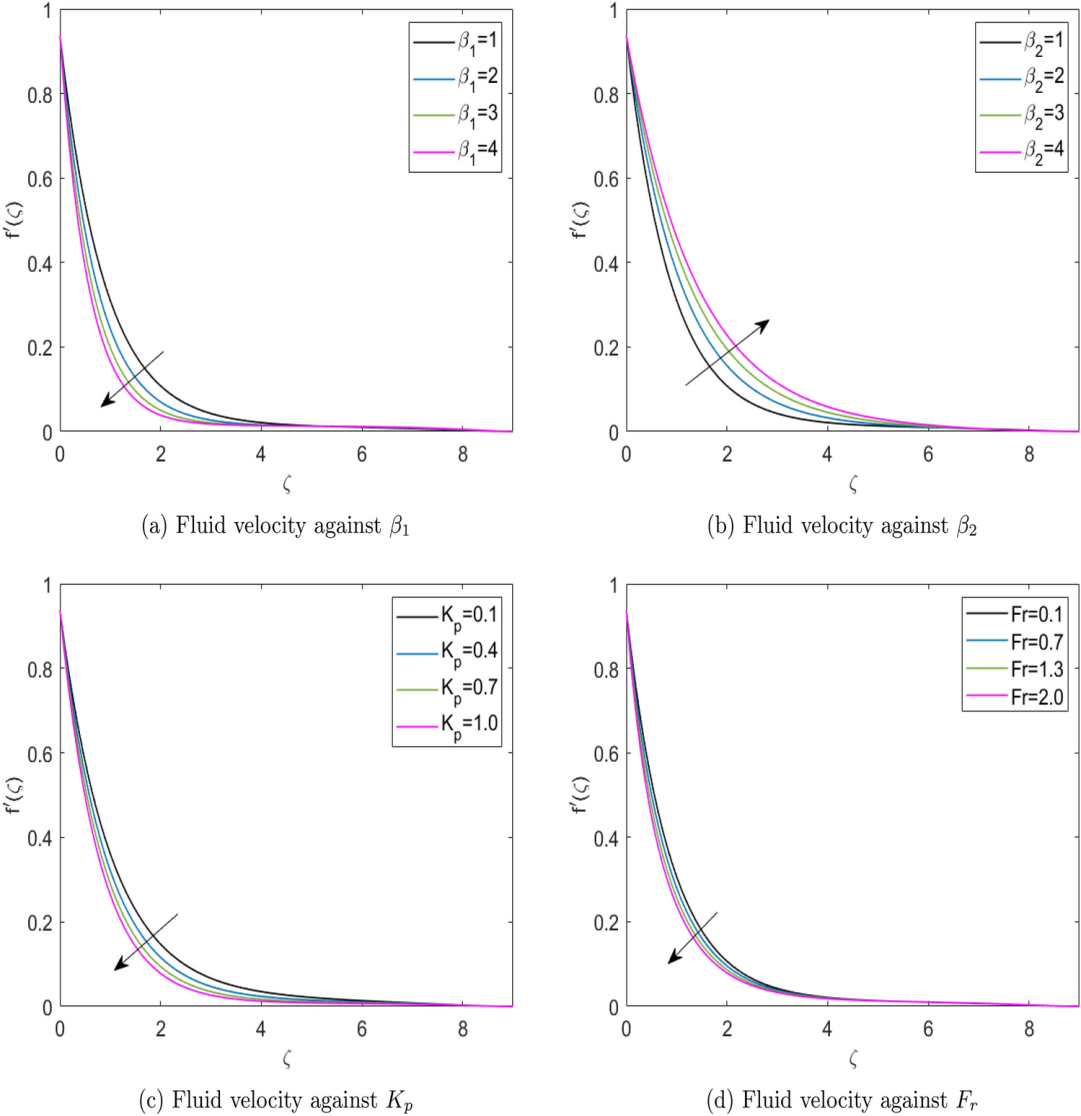
Fluid velocity against diverse physical parameters.

Figures 6a–d show the results of fluid velocity for different physical parameters in the form of graphs. Figure 6a depicts how Deborah number ($\beta _1$) affects the fluid velocity. This figure reports that when $\beta _1$ increases, fluid velocity decreases. A fluid flow with a lower Deborah number behaves more like a fluid material, and a higher Deborah number flow behaves more like a solid structure. Hence, the fluid velocity worsens as $\beta _1$ increases. Figure 6b depicts the fluid’s velocity variations for retardation time parameter ($\beta _2$). The flow field’s boundary layer escalates as the retardation time parameter ($\beta _2$) increases. The increase of $\beta _2$ means higher retardation time which is due to the more considerable momentum of fluid particles in the boundary layer, and so does the velocity profile enhancement in the flow field. Velocity profile for the diverse values of the Darcy number ($K_p$) and the Forchheimer number (*Fr*) are depicted in Fig. 6c and d. These Figures reveal that the escalation in the $K_p$ and *Fr* diminishes the velocity profiles. This is due to the effect of the resistance generated by the porous structure of the permeable medium.

**Fig. 7:**
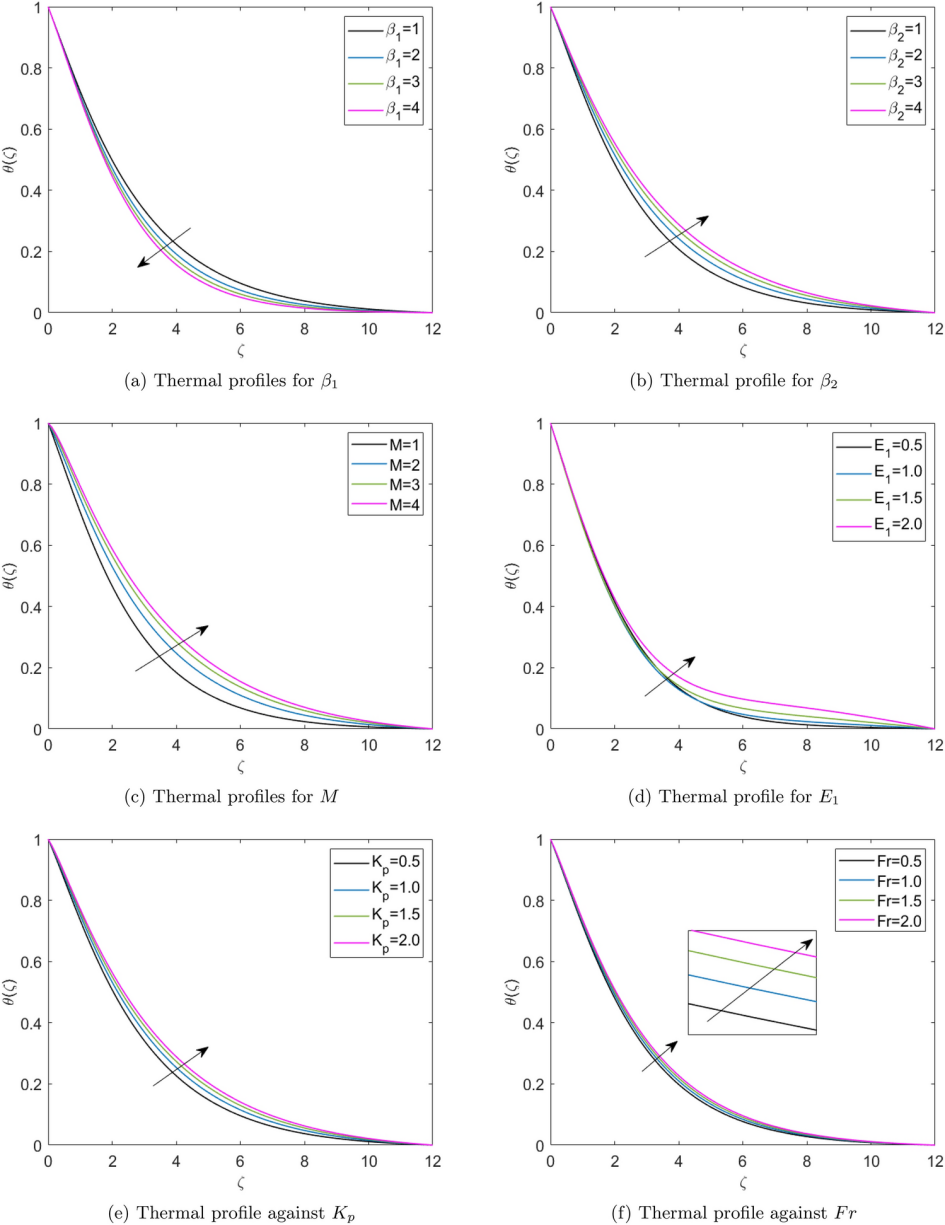
Thermal profiles for the diferent physical parameters.

**Fig. 8:**
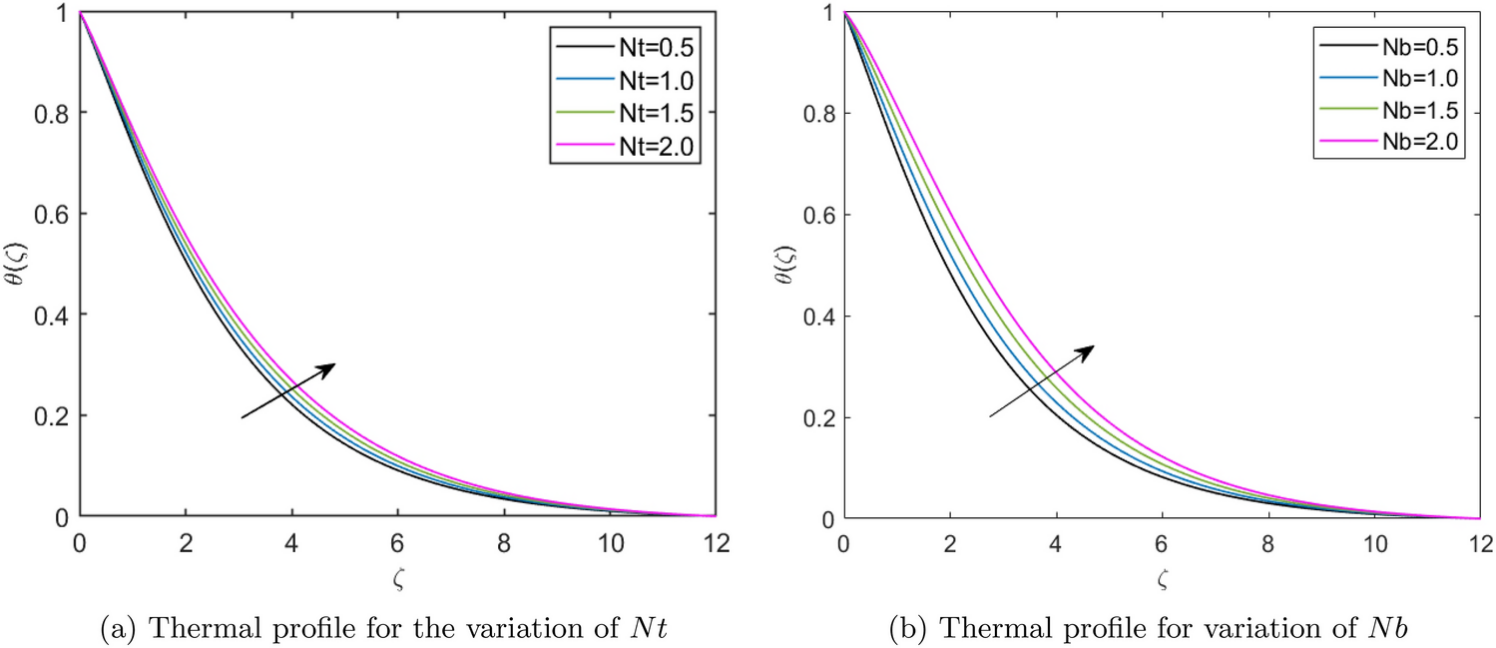
Thermal profiles for different physical parameters.

Figure 7a reports the thermal profiles resulting from the Deborah number ($\beta _1$). This Figure presents that the thermal profiles in the boundary layer are reduced with the higher Deborah values, as the $\beta _1$ is the ratio of relaxation to retardation time. This comprises both the material’s elastic and viscous properties. The material behaves more fluid-like at lower Deborah numbers, with a Newtonian viscous flow. At more significant Deborah numbers, the material behavior shifts to a non-Newtonian domain when elasticity dominates, and solidlike behavior emerges. Therefore, thermal profiles reduce with the higher Deborah number. Figure 7(b) picturizes the thermal profiles for the varying values of retardation time parameter ($\beta _2$). This Figure presents that the thermal profiles enhance for the greater values of the retardation time parameter. A fluid flow with lower retardation time offers more vital viscous forces, which weakens the diffusion of the fluid particles in the boundary layer. Therefore, thermal profiles are higher for the higher reatardation time parameter and are lesser for the lower retardation time parameter. Figure 7(c) depicts fluid thermal profiles for the *M*. A higher value of *M* improves the fluid’s temperature because a higher magnetic field strength provides a more vital Lorentz force, enhancing the thermal profiles. Figure 7(d) presents the influence of the electric field parameter ($E_1$) on the non-dimensional temperature profiles. The thermal profiles exhibit reduction up to a fixed point of the similarity variable ($\zeta$) but subsequently shift their behavior and portray an enhancing effect as they approach the wall of elongated surface. Figure 7(e) illustrates the effect of Darcy number ($K_p$) on the thermal profiles. This Figure declares that the higher Darcy number escalates the thermal profiles in the fluid flow. The Darcy number represents the porosity of the material, and the solid layers form the porous medium which resists the fluid flow and results in the better mixing of the fluid layers, further enhancing the thermal boundary layer. Figure 7(f) reports the thermal profiles affected by the Forchheimer number (*Fr*). This Figure reports that the thermal profile enhances the value of the Forchheimer number. Forchheimer number represents the permeability of the porous material. Permeability is a feature of porous materials that indicates the ability of fluids to pass through them. A highly permeable porous material allows fluids to flow more easily than the less permeable porous material. Therefore, high permeability means more effortless fluid movement in the porous layers of the porous medium, which results in effective thermal transmission in the boundary layer. Figure 8(a) indicates the thermal profiles obtained for the varying default values of the thermophoretic diffusion parameter (*Nt*). This Figure illustrates that an improved thermophoresis parameter escalates the thermal profiles. Thermophoretic diffusion is the response of the fluid molecules to push nanoparticles from the hotter part to the colder part resulting in the thermal gradients. Hence, the improved movement of nanoparticles in the fluid layers enhances the thermal profiles in the boundary layer. Figure 8(b) displays the effect of *Nb* on the thermal profiles. This Figure reveals that the escalation of *Nb* enhances the thermal profiles. As the Brownian motion is the random or zigzag movement of nanoparticles in the fluid. Hence, the more zigzag motion of the nanoparticles results in improved mixing of fluid layers and enhanced momentum of fluid molecules, which escalates the heat transfer in fluid flow.

### Physical quantity of engineering interest

Nusselt number ($$N u_{x}$$)

6.1 $$\begin{aligned} Nu_{x} =\frac{(x+b) q_{s}}{\kappa _{hnf}\left( T-T_{\infty }\right) }, \quad q_{s}=-\left. \bigg (\kappa _{hnf}+\frac{16 \sigma T_\infty ^3}{3 \kappa ^*}\bigg ) \frac{\partial T}{\partial y}\right| _{y=A(x+b)^{\frac{1-n}{2}}}, \end{aligned}$$

Above quantity in non-dimensional form:

6.2 $$\begin{aligned} Re_{x} ^{-1 / 2} N u_{x}=-\left. \sqrt{\frac{n+1}{2}} (\varepsilon _6+Nr)\theta ^{\prime }(\zeta )\right| _{\zeta =0}, \quad \end{aligned}$$

**Fig. 9:**
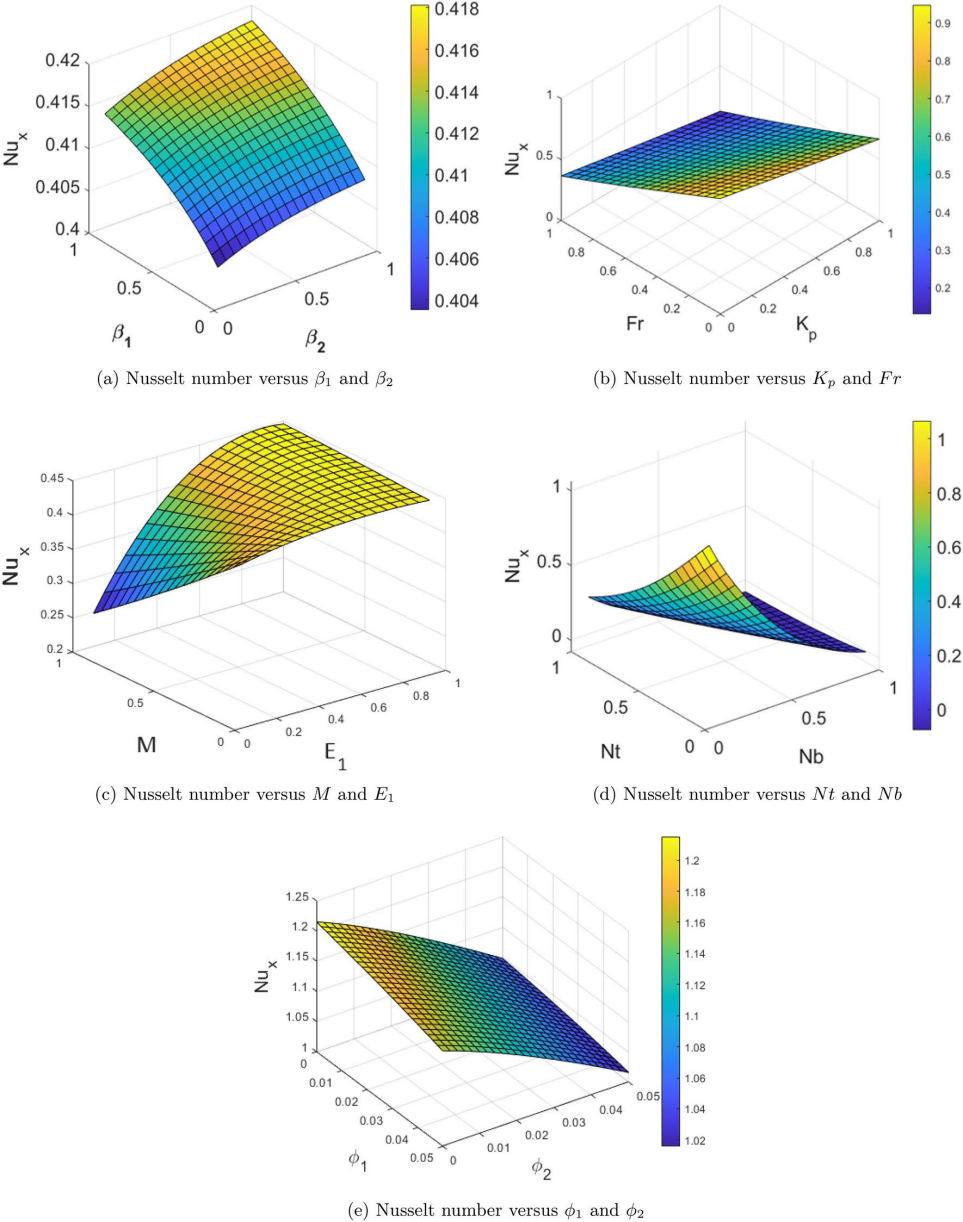
Outcomes of the Nusselt numbers against diverse physical parameters.

Numerical outputs for the Nusselt number ($Nu_x$) corresponding to the various effective physical parameters are presented in terms of the surface plots in the Fig. 9a–e. Figure 9(a) portrays the variation in $Nu_x$ due to the physical parameters $\beta _1$ and $\beta _2$. From this Figure it is seen that the escalation in both the parameters $\beta _1$ and $\beta _2$ enhances the $Nu_x$. Figure 9(b) illustrate the effect of physical parameters $K_p$ and $F_r$ on the $Nu_x$. This Figure reveals that the escalation of physical parameters $K_p$ and $F_r$ diminishes the $Nu_x$. Figure 9(c) depicts the variation in $Nu_x$ due to the physical parameters *M* and $E_1$. This Figure reports that the increase in magnetic field reduces the $Nu_x$ however increase in the $E_1$ escalates the Nusselt number. Effect of the physical parameters *Nt* and *Nb* on $Nu_x$ is presented in the Fig. 9(d). This Figure declares that $Nu_x$ decelerates the with the enhancement in both parameters *Nt* and *Nb*. $Nu_x$ results for the variation in nanofluid concentrations $\phi _1$ and $\phi _2$ are shown in the Fig. 9(e). As per this Figure, it is noted that the enhancement in nanofluid concentrations $\phi _1$ and $\phi _2$ decreases the $Nu_x$. This means that the conductive heat transfer becomes more dominant for the higher nanofluid concentrations.

### Entropy generation modeling

Expression of entropy generation is formulated as^64^:

6.3 $$\begin{aligned} E_g= & \frac{1}{(T_{\infty })^{2}}\bigg ({\kappa _{hnf} +\frac{16 \sigma ^* T_\infty ^3}{3 \kappa (\rho c_p)_{hnf}}}\bigg )\bigg (\frac{\partial T}{\partial y}\bigg )^2+\frac{\mu _{hnf}}{T_{\infty }(1+\lambda _2)} \bigg [\bigg (\frac{\partial u}{\partial y}\bigg )^{2}+\lambda _{1}\bigg (u \frac{\partial u}{\partial y} \frac{\partial ^{2} u}{\partial x \partial y}+v\frac{\partial u}{\partial y} \frac{\partial ^{2} u}{\partial y^{2}}\bigg )\bigg ] + \frac{\sigma _{hnf}}{(T_{\infty })^{2}}(uB-E)^2 \nonumber \\ & +\frac{RD_b}{C_{\infty }}\bigg (\frac{\partial C}{\partial y}\bigg )^2 +\frac{RD_b}{T_{\infty }}\bigg (\frac{\partial C}{\partial y}\bigg )\bigg (\frac{\partial T}{\partial y}\bigg )+\frac{RD_b}{N_{\infty }}\bigg (\frac{\partial N}{\partial y}\bigg )^2 +\frac{RD_b}{T_{\infty }}\bigg (\frac{\partial N}{\partial y}\bigg )\bigg (\frac{\partial T}{\partial y}\bigg ). \end{aligned}$$

Formulation of the expression into non-dimensional form:

6.4 $$\begin{aligned} N_{s}= & (1 +\frac{4}{3}Rd)\delta {\theta ^\prime }^2+A_1 \frac{PrEc}{(1+\beta _1)}\bigg [\bigg (\frac{\partial ^2 f}{\partial \zeta ^2}\bigg )^2+\beta _2 \bigg [\bigg (\frac{\partial ^2 f}{\partial \zeta ^2}\bigg )^2\frac{\partial f}{\partial \zeta }+\xi \frac{\partial ^2 Q}{\partial \zeta ^2}\frac{\partial f}{\partial \zeta }\frac{\partial ^2 f}{\partial \zeta ^2}-\frac{n+1}{2}\bigg (\frac{2\xi }{n+1} Q+f+\frac{n-1}{n+1}\zeta \frac{\partial f}{\partial \zeta }\bigg ) \nonumber \\ & \frac{\partial ^2 f}{\partial \zeta ^2}\frac{\partial ^3 f}{\partial \zeta ^3} \bigg ]\bigg ] +A_3MPrEc(f^\prime -E_1)^2+L \theta ^\prime \phi ^\prime +L^*\frac{1}{\delta \delta _N}{\chi ^\prime }^2+L\frac{\delta _1}{\delta }{\phi ^\prime }^2+L^*\theta ^\prime \chi ^\prime . \end{aligned}$$

**Fig. 10:**
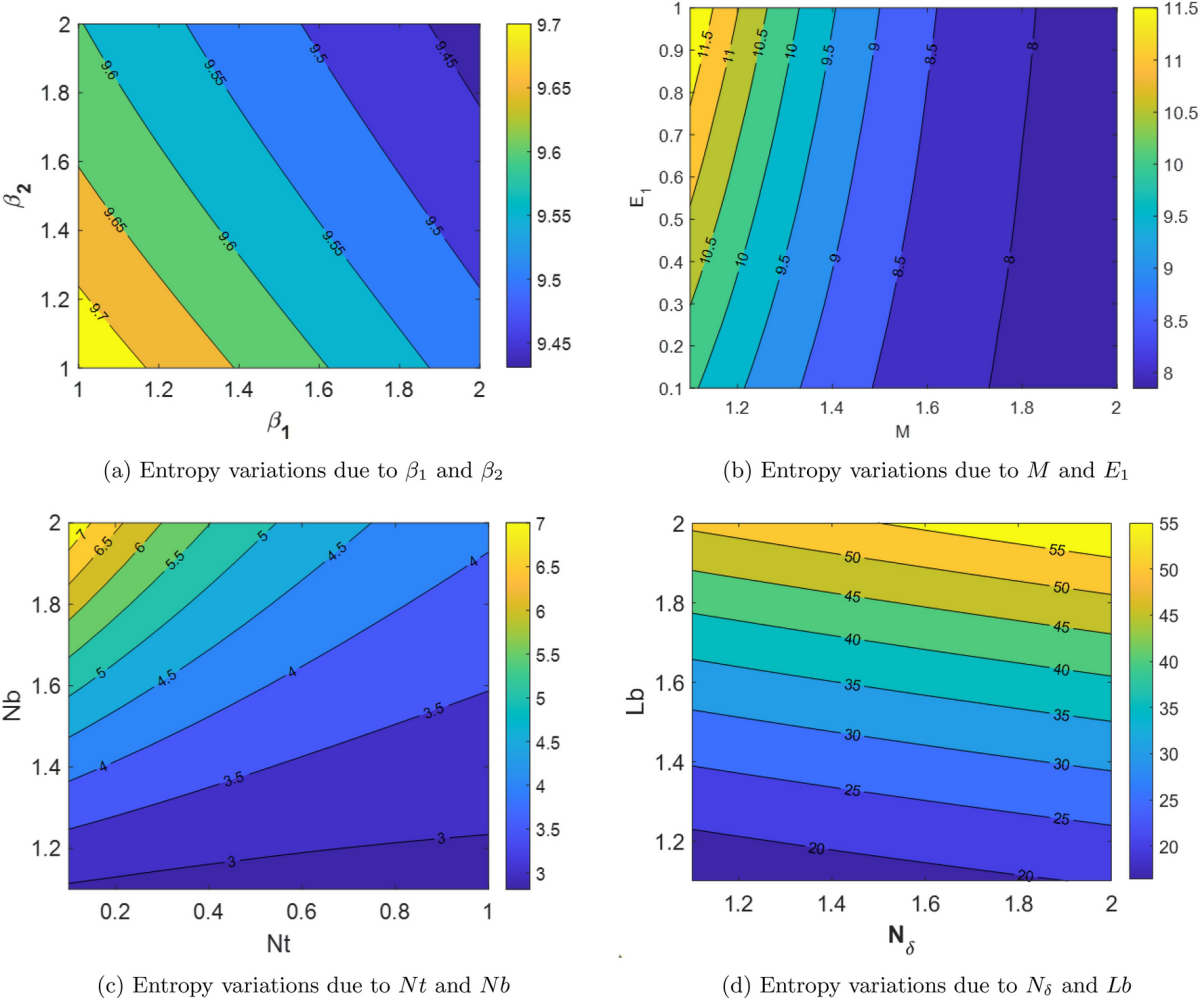
The influence of diverse physical parameters on entropy generation.

Entropy generation ($N_s$) results due to diverse physical parameters are shown in the Figures 10(a)-10(d). Figure 10(a) depicts the $N_s$ results corresponding to the physical parameters $\beta _1$ and $\beta _2$. This shows that the $N_s$ reduces along the increasing values of $\beta _1$ and $\beta _2$. The Fig. 10(b) demonstrate the influence of physical parameters *M* and $E_1$ on the $N_s$. This Figure reports that the $N_s$ enhances for the improvement of parameter $E_1$. And, the $N_s$ reduces with an increment in the parameter *M*. Variation in the $N_s$ due the influence of the physical parameters *Nt* and *Nb* is presented in the Fig. 10(d). As per this Figure it is noticed that the augmentation in the physical parameter *Nt* decreases the $N_s$ while the escalation of parameter *Nb* enhances $N_s$. Figure 10(d) depicts the effect of bioconvection Lewis number (*Lb*) and microorganism concentration difference parameter ($\delta _N$) on the entropy formation. From this Figure it is observed that the escalation in both the physical parameters $\delta _N$ and *Lb* enhances the entropy generation.

## Conclusions

This investigation performs the study of heat transfer by thermophoresis and Brownian motion of nanoparticles influenced with the suspended gyrotactic microorganisms through Darcy Forchheimer porous medium due to Jeffrey’s electro-magnetohydrodynamic hybrid nanofluid flow for solar thermal collectors. Mathematical modeling of the governing equations uses the conservation principle of mass, momentum, energy, concentration, and microorganism concentration. Appropriate non-similar variables are introduced to the governing equations. These variables simplifies the governing equations to the dimensionless ODEs. The dimensionless equations are numerically simulated using Cash and Carp methodology. The artificial neural network is designed for the developed mathematical model by the Levenberg Marquardt algorithm. Numerical findings corresponding to the input parameters influencing the fluid flow and heat transfer are mentioned below:

- Thermal profiles for the Deborah number ($\beta _1$) reduces with growing values of Deborah number ($\beta _1$).
- Thermal profiles corresponding to the Deborah number ($\beta _2$) enhances with the escalation of retardation time parameter ($\beta _2$).
- Thermal profiles shows dual behavior corresponding to varying the electric field parameter ($E_1$), thermal profiles first decreases and then increases towards the boundary layer.
- Thermal profiles with respect to Hartmann number (*M*) enhances with escalating values of Hartmann number.
- Thermal profiles enhances for the escalation in the Darcy number (*Da*) and Forchheimer number (*Fr*).
- An increase in thermophoretic diffusion parameter (*Nt*) and Brownian motion parameter (*Nb*) escalates the thermal profiles.
- Nusselt number enhances with the escalation in both the Deborah number ($\beta _1$) and retardation time parameter ($\beta _2$).
- Nusselt number decelerates with the enhancement in the Darcy number and Forchheimer number.
- Nusselt number is higher for the larger electric field parameter and Nusselt number lesser for the larger Hartmann number.
- Nusselt number decelerates with the enhancement in thermophoretic diffusion parameter and Brownian motion parameter.
- Higher values of the nanofluid concentrations ($\phi _1$, and $\phi _2$) diminishes the Nusselt number.
- Entropy generation reduces with an enhancement in Deborah numbers ($\beta _1$) and retardation time parameter ($\beta _2$).
- Entropy generation decreases along the enhancement in thermophoretic diffusion parameter (*Nt*) and entropy generation increases along the Brownian motion parameter (*Nb*).
- Entropy generation reduces along the increase in Hartmann number and entropy generation increases along the escalation of thermophoretic diffusion parameter (*Nt*).
- Entropy generation escalates for the increase in microorganism concentration difference parameter ($N_\delta$) and the bioconvection Lewis number (*Lb*).

The concentrated solar power technology, specifically the parabolic trough collector, is used for efficient solar energy conversion. This energy can be converted into heat or electricity for diverse practical applications like electricity generation, industrial heating processes, and space heating or cooling. The immediate focus of this attempt is likely to improve the performance and efficiency of parabolic trough solar collectors. This could involve reducing radiant energy loss, enhancing the overall design, increasing energy capture, or improving the thermal storage systems associated with concentrated solar power technology. Researchers and scientists are primarily interested in this application to make concentrated solar power technology a more cost-effective and environmentally friendly energy source, particularly in regions with abundant sunlight.

### Future scope

This study can be extended to the performance evaluation of the tri or tetra hybrid nanofluid flows by the use of advanced phase change materials having high radiation absorption properties. This study can also be done for the turbullence modeling with multiphase simulations of heat transfer by inserting the different shapes of turbulators in the receiver tube.

## Data Availability

All data generated or analysed during this study are included in this published article.

## List of symbols

*C*: Concentration of nanoparticles
$C_{p}$: Specific heat at constant pressure
$C_s$: Concentration at the surface of the wall
$$C_\infty$$: Ambient concentration
$D_B$: Brownian diffusion coefficient
$D_T$: Thermophoresis diffusion coefficient
$E_1$: Electric field parameter
$$E_c$$: Eckert number
$$f^\prime$$: Dimensionless velocity
*L*: Diffusion parameter
*Lb*: Bioconvection Lewis number
*Le*: Lewis number
$L^*$: Bioconvection diffusion parameter
*M*: Magnetic field parameter
*N*: Concentration of microorganisms
$N_\infty$: Ambient concentration of microorganisms
*Nt*: Thermophoresis diffusion parameter
*Nb*: Brownian diffusion parameter
$Nn_x$: Local density of microorganisms
$$Nu_x$$: Nusselt number
*Pe*: Peclet number
*Pr*: Prandtl number
$$T_\infty$$: Ambient temperature
$T_s$: Temperature at the surface of the wall
$u$: Velocity component in x-direction
$v$: Velocity component in y-direction
$U_s$: Stretching sheet velocity

## Greek symbols

$$\alpha$$: Thickness parameter
$$\alpha _1$$: Thermal diffusivity
$\beta _1$: Relaxation and reatardation time ratio parameter
$\beta _2$: Retardation time parameter
$\delta$: Temperature difference ratio
$\delta _1$: Concentration difference ratio
$N_\delta$: Microorganism concentration difference ratio
*k*: Thermal conductivity
$\lambda _1$: Relaxation and reatardation time ratio
$$\lambda _2$$: Retardation time
$\nu_f$: Kinematic viscosity of fluid
$\xi$: Non-similar variable in x-direction
$$\psi$$: Stream function
$\mu_f$: Dynamic viscosity of fluid
$\mu_{hnf}$: Dynamic viscosiy of the hybrid nanofluid
$\nu_{hnf}$: Hybrid nanofluid kinematic viscosity
$$\phi$$: Dimensionless concentration
$\rho_{hnf}$: Density of hybrid nanofluid
$$\sigma_{hnf}$$: Electrical conductivity
$\tau _ s$: Shear stress at the wall surface
$$\theta$$: Dimensionless temperature
*k*_*f*_: Thermal conductivity of fluid
*X*: Dimensionles concentration of microorganisms
$\varsigma$: Non similar variable in y-direction
$\phi _1$: Concentration of Silver nanoparticles
$\phi _2$: Concentration of Graphene nanoparticles
